# Metabolomic Insights into the Antimicrobial Effects of *Metschnikowia* Yeast on Phytopathogens

**DOI:** 10.3390/molecules30153268

**Published:** 2025-08-04

**Authors:** Zofia Perek, Sumi Krupa, Joanna Nizioł, Dorota Kręgiel, Tomasz Ruman, Beata Gutarowska

**Affiliations:** 1 Department of Environmental Biotechnology, Lodz University of Technology, Wolczanska 171/173, 90-530 Lodz, Poland; dorota.kregiel@p.lodz.pl (D.K.); beata.gutarowska@p.lodz.pl (B.G.); 2 Interdisciplinary Doctoral School, Lodz University of Technology, Zeromskiego 116, 90-924 Lodz, Poland; 3 Faculty of Chemistry, Rzeszow University of Technology, Powstancow Warszawy 6, 35-959 Rzeszow, Poland; d605@stud.prz.edu.pl (S.K.); jniziol@prz.edu.pl (J.N.); tomruman@prz.edu.pl (T.R.)

**Keywords:** *Metschnikowia pulcherrima* yeast, antimicrobial activity, metabolomics, phytopathogens

## Abstract

One of the most important features of *Metschnikowia pulcherrima* is its strong antimicrobial activity against phytopathogens, which makes it a suitable candidate for use in biocontrol during crop cultivation. However, the mechanisms of its antimicrobial activity are not currently well understood. In this study, we used metabolomic methods to investigate the possible mechanisms of antimicrobial activity by *M. pulcherrima* against phytopathogenic fungi. First, we tested the antimicrobial activity of five selected isolates against eleven phytopathogenic molds. Based on the results, selected yeast–pathogen co-cultures were cultivated on liquid and solid media. The supernatants from the liquid co-cultures were analyzed using the UHPLC-QToF-UHRMS and MS/MS methods. Co-culture growth on solid agar media was examined using the LARAPPI/CI MSI method. The yeast exhibited strong antagonism toward the mold phytopathogens. The LARAPPI/CI MSI method revealed the presence of various compounds with potential antifungal activity. The complex UHPLC-QToF-UHRMS analysis confirmed that the metabolic response of *M. pulcherrima* depends on specific yeast–pathogen interactions.

## 1. Introduction

*Metschnikowia pulcherrima* is an unconventional yeast with broad adaptability and potential applications for bioprotection. The yeast naturally occurs on fruits and flowers, nectar, and tree sap; therefore, it may also be part of the insect microbiota [1,2,3]. *M. pulcherrima* exhibits a range of features that are useful in biotechnology [2,3,4,5,6]. Notably, they can be used in fermentation processes to enhance the organoleptic properties of wines or meads [1,7,8]. They can also be used in the production of microbial oils, due to their ability to accumulate fatty acids within cells [9]. One of the most important characteristics of *M. pulcherrima* is its antimicrobial activity, which makes it suitable for use in biocontrol processes to protect plants from phytopathogens [2,10].

Phytopathogens are the etiological agents of plant diseases, which cause reductions in both the quality and quantity of crops. It is estimated that phytopathogens cause up to 15% of the losses in global crop production each year [11]. Molds, including genera *Fusarium*, *Venturia*, *Alternaria*, and *Botrytis*, are particularly dangerous, and are responsible for 70–80% of all plant diseases [11]. Additionally, fungal phytopathogens from the genus *Aspergillus* and *Penicillium* cause postharvest diseases, which also lead to enormous losses [12,13,14,15]. Bacterial phytopathogens are also dangerous; for example, *Pseudomonas syringae* can reduce crop yields by up to 20% and cause plant death [16].

Current strategies for combating phytopathogens have negative environmental impacts, which is why new methods for preventing plant diseases are being sought [11,12,17]. Chemical pesticides, which are the most common method of plant protection, work by disrupting basic cellular processes. Widely used fungicides include azoxystrobin, diphenylamine, propiconazole, tebuconazole, and etridiazole [18]. Unfortunately, these agents accumulate in the soil and affect organisms that are not the target group, disturbing the homeostasis of the environment [18,19]. An eco-friendly bioproduct containing *Metschnikowia pulcherrima* cells could provide an alternative [5,20,21,22].

The main and most well-known mechanism of antimicrobial activity by *M. pulcherrima* yeast is the production of pulcherrimin, a red pigment. These yeasts produce pulcherriminic acid, which chelates iron ions and transforms into pulcherrimin. This mechanism is based on iron sequestration, which makes this element unavailable for other microorganisms, including fungi. The absence of iron ions in the environment thus inhibits mycelium growth [10,23]. Other factors that contribute to the antimicrobial activity of *M. pulcherrima* include competition for nutrients, the production of lytic enzymes, production of organic acids, and even modulation of the immune response in plants [4,5,15,24].

*M. pulcherrima* has been found to have antifungal effects against molds including *Botrytis cinerea* [10,20], *Penicillium expansum* [20,22], *Monilia* sp. [20], *Fusarium oxysporum*, *F. sambucinum*, *Alternaria alternata*, *A. solani*, *A. tenuissima*, *Colletotrichum coccodes*, *Rhizoctonia solani*, *Phoma exigua* [5], *Aspergillus niger*, *Rhizopus oryzae*, and *Verticillium cinnabarinum* [25]. However, the antimicrobial mechanisms of *M. pulcherrima* against plant phytopathogens are not fully understood. There are limited data on this topic in the literature, which focuses mainly on individual mechanisms. *M. pulcherrima* can also inhibit bacterial growth, although its antibacterial activity is much weaker. For these reasons, we decided in this study to focus on understanding the antifungal mechanisms of *M. pulcherrima*, using metabolomic methods [5].

Metabolomic methods allow for the detailed examination and profiling of microbial metabolites. Using these technologies, it is possible to select strains of microorganisms that exhibit the most useful features such as, in the development of yeast biopreparations, the ability to protect plants against phytopathogens. Ultra-high-performance liquid chromatography combined with mass spectrometry (UHPLC-QToF-UHRMS) can be a valuable tool for studying the metabolites produced by microorganisms [26,27]. Laser ablation remote atmospheric pressure photoionization/chemical ionization mass spectrometry imaging (LARAPPI/CI MSI) is an innovative method that allows for the mapping of various types of chemical compounds in samples (e.g., human or plant tissues, agarose gel plates, etc.) [28]. This is useful for studying the mechanisms of antimicrobial activity, while also allowing for the detection of chemical compound gradients and the determination of the concentration at which microbial growth inhibition occurs [27].

The purpose of this study was to assess the metabolite profiles of *M. pulcherrima* yeast in the presence of phytopathogens, using metabolomic methods (UHPLC-QToF-UHRMS and LARAPPI/CI MSI). This study is the first to report the metabolomic profiling of co-cultures of *M. pulcherrima* yeast and phytopathogenic fungi using the LARAPPI/CI-MSI method. It also employed innovative methods for the metabolomic analysis of the culture supernatants and to investigate the multiple interactions between phytopathogenic fungi and *Metschnikowia* yeasts.

Two research hypotheses guided this study: 1. The yeast *Metschnikowia pulcherrima* changes its antimicrobial metabolic profile depending on its interaction with different species of fungal phytopathogens; 2. *Metschnikowia pulcherrima* has a broad spectrum of antimicrobial mechanisms that have not yet been described in the scientific literature and produces numerous chemical compounds that inhibit the growth of phytopathogenic fungi. The scope of this study included examining the antimicrobial activity of five *M. pulcherrima* strains in two forms: cell suspensions and cell-free post-culture liquids. The co-cultivation of yeast with phytopathogens was carried out in liquid media, followed by analysis using UHPLC-QToF-UHRMS. Additionally, co-cultures on solid media were analyzed using the LARAPPI/CI-MSI method. The results enable the identification of the most effective yeast strains and provide deeper insights into the mechanisms underlying the antifungal activity of *M. pulcherrima*.

## 2. Results and Discussion

### 2.1. Antifungal Activity

**Figure 1:**
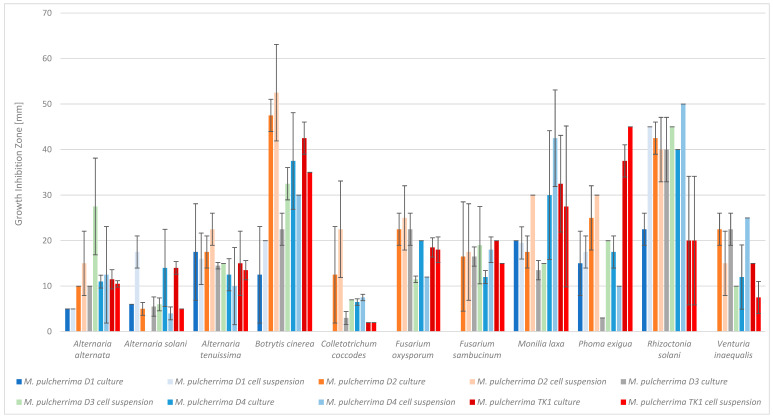
Antimicrobial activity of *Metschnikowia pulcherrima* cultures and cells suspensions against phytopathogens, measured as growth-inhibition zones. *M. pulcherrima* TK1 showed a strong inhibition of all tested phytopathogens. However, the largest inhibition zones were observed for D2 and D4. The weakest yeast strain was D1, followed by D3. Statistical analysis showed that, depending on the yeast strain, the inhibition zones differed significantly for the following phytopathogens: *Alternaria solani*, *Botrytis cinerea*, *Colletotrichum coccodes*, *Fusarium oxysporum*, *Phoma exigua*, *Rhizoctonia solani*, and *Venturia inaequalis* (both the yeast culture and the cell suspension). No statistically significant differences in inhibition zones were observed for the following phytopathogens: *A. alternata*, *A. tenuissima*, *F. sambucinum*, and *M. laxa*, (Figure 1, Table S2). *M. pulcherrima* D2, D4, and TK1 were identified as the most effective at inhibiting the growth of phytopathogens. Therefore, these strains were selected for further research.

The cultures of *M. pulcherrima* strains D2, D3, D4, and TK1 inhibited the growth of all tested phytopathogens (Figure 1, Table S2) on PDA medium. The culture of strain D1 did not inhibit the growth of the following four tested phytopathogens: *C. coccodes*, *F. oxysporum*, *F. sambucinum*, and *V. inaequalis*. The growth inhibition zones caused by the *Metschnikowia* culture varied and ranged from 2 mm to 47.5 mm (Table S2). The cell suspension of *M. pulcherrima* D3, D4, and TK1 inhibited the growth of all tested phytopathogens. The cell suspension of strain D2 did not inhibit the growth of *A. solani*, and the suspension of strain D1, similarly to the culture of this strain, did not inhibit the growth of *C. coccodes*, *F. oxysporum*, *F. sambucinum*, or *V. inaequalis.* The results were varied and ranged from 2 mm to 52.5 mm. The smallest inhibition zones were observed for the phytopathogens *C. coccodes* (from 2 mm to 22.5 mm) and *A. solani* (from 4 mm to 17.5 mm. The largest zones were observed for: *B. cinerea* (from 12.5 mm to 52.5 mm), *R. solani* (from 20 mm to 50 mm) and *M. laxa* (from 15 mm to 40 mm). For the remaining phytopathogens, growth inhibition was observed within the following ranges: *A. alternata* (from 5 mm to 27.5 mm), *A. tenuissima* (from 10 mm to 22.5 mm), *F. oxysporum* (from 11.5 mm to 25 mm), *F. sambucinum* (from 12 mm to 20 mm), *P. exigua* (from 3 mm to 45 mm), and *V. inaequalis* (from 7.5 mm to 25 mm) (Table S1). A statistical analysis of the results was performed (Tukey’s post hoc test at a significance level of 0.05). Statistically different samples are marked with different letters within the same phytopathogen (a, b, c…) in Table S2.

It was observed that both *M. pulcherrima* yeast cultures and yeast cell suspensions inhibited the growth of phytopathogens, which confirms the results of Spadaro et al. [20]. Steglińska et al. [5] studied the antimicrobial activity of *M. pulcherrima* against potato phytopathogens, including *A. alternata*, *A. solani*, *A. tenuissima*, *C. coccodes*, *F. oxysporum*, *F. sambucinum*, *P. exigua*, and *R. solani*. Similar results were obtained, especially for strain TK1 against *F. sambucinum* (15 mm). Differences for *A. alternata*, *A. solani*, *R. solani*, and *C. coccodes* were minor (2–3 mm). Larger discrepancies were noted for *F. oxysporum* (18 mm vs. 7 mm), *A. tenuissima* (13.5 mm vs. 8 mm), and *P. exigua* (45 mm vs. 5 mm). These differences may result from variations in yeast culture conditions, such as media or incubation time. Inhibition of *Botrytis cinerea* growth by *M. pulcherrima* yeast has been widely reported, including by Pawlikowska et al. [25], Sipiczki [10], and Agarbati et al. [29]. Pawlikowska et al. [25] studied the antimicrobial activity of *M. pulcherrima* strains D1–D4 against *A. alternata* and *B. cinerea*, reporting inhibition zones in the range of 7–15 mm. In our study, similar results were observed for strains D1, D2, and D4, while strain D3 showed a significantly larger zone (27.5 mm). For *B. cinerea*, much larger inhibition zones were noted compared to Pawlikowska et al., likely due to methodological differences [25]. Sipiczki tested the effect of *M. pulcherrima* on the germination and growth of *B. cinerea*, and observed the inhibition of both the germination and growth of *B. cinerea* [10]. Agarbati et al. [29] observed that all tested *M. pulcherrima* strains inhibited the growth of gray mold [29]. These results are consistent with the findings of the present study. Ruiz-Moyano [30] reported similar results, with *M. pulcherrima* inhibiting *Monilia laxa* growth by 45–66% in vitro. In our study, strains D1, D2, D3, D4, and TK1 also showed moderate to strong inhibition. The antimicrobial activity of *M. pulcherrima* against *V. inaequalis* has been described only under in vivo conditions on apples, by Urazova et al. [31].

No growth of phytopathogens was observed in co-cultures of *M. pulcherrima* yeast with molds on MEB medium (Table S3 in Supplementary Materials). Mold growth was observed only in liquid cultures, when the phytopathogens were grown as monocultures. In the co-cultures of *M. pulcherrima* yeast and molds, only slight turbidity was observed, characteristic of a typical yeast culture. The control cultures of the phytopathogens were dense, and a pellet formation was observed (*A. alternata*) or large, solid, mycelium biomasses (*R. solani*, *B. cinerea*, *M. laxa*). This may indicate that in the co-cultures in MEB liquid medium, the yeast took over the environment, which did not allow the development of phytopathogens. This contrasts with the situation observed in solid medium (PDA), where phytopathogen growth occurred, but was limited. This was likely due to the more favorable conditions for yeast proliferation in the liquid medium (e.g., better oxygenation and access to nutrients, shorter generation time), which support rapid yeast growth and the production of metabolites that inhibit phytopathogen development. The TK1 yeast strain showed the most growth, followed by strain D4, while strain D2 exhibited the least growth. Statistical analysis showed that the greatest differences in yeast growth were observed for strain D2, D4 (Table S3).

This study is the first to describe the results of co-culturing *M. pulcherrima* with the tested phytopathogens (*R. solani*, *B. cinerea*, *M. laxa*, *A. alternata*) on the selected media. Although there are no data in the literature regarding co-cultures of *Metschnikowia* yeasts and the studied phytopathogens in MEB medium, there are scientific reports in which *M. pulcherrima* was observed to inhibit the growth of other microorganisms on a liquid medium. For example, Türkel et al. [32] observed the complete inhibition of pathogens including *P. expansum*, *Fusarium* sp., and *Rhizopus* sp. by *M. pulcherrima* yeast in grape juice. Aragno et al. [33] conducted similar studies by co-culturing *Metschnikowia* yeast and with the bacteria *Gluconobacter oxydans* and yeast *Brettanomyces bruxellensis* in grape juice. It was observed that the *Metschnikowia* yeast slowed down the growth of the other microorganisms. Most studies focusing on the antimicrobial activity of *M. pulcherrima* focus on confirming its effectiveness on solid media or under in vivo conditions [5,10,28,29,30,31].

### 2.2. Metabolomic Analysis

#### 2.2.1. Laser Ablation Remote Atmospheric Pressure Photoionization/Chemical Ionization Mass Spectrometry Imaging (LARAPPI/CI MSI)

This study is the first to apply the LARAPPI/CI MSI method to identify chemical compounds in co-cultures of *Metschnikowia pulcherrima* and phytopathogens grown on solid PDA medium. This method enables both the identification of compounds and visualization of their spatial distributions, revealing their ability to diffuse through the substrate. A total of 160 compounds were detected, including 93 from primary metabolism and 65 from secondary metabolism (Table S4). Selected metabolites showing differential production in co-cultures versus pure cultures are listed in Table 1. The resulting images (Table 2; Tables S5–S7 in the Supplementary Materials) reveal concentration gradients of individual compounds, for example, acetic acid and pyruvaldehyde produced by strain D2 with *A. alternata* and strain D4 with *M. laxa* (Table 2).

The compounds detected in this study include several with known antimicrobial properties, including indole, indole-3-carboxylic acid, 3-methylindole, 3,5-dimethoxyphenol, cinnamic acid, hydrocinnamic acid, 4-methylcinnamic acid, *m*-coumaric acid, *p*-anisic acid, azelaic acid, perillic acid, syringic acid, phenylacetic acid, lactic acid, oleic acid, and pulcherriminic acid (Table 1). Cinnamic acid, a compound with notable antimicrobial activity, was identified in co-cultures of strains D2 and D4. This compound exhibits antiviral, antibacterial, and antifungal properties [34,35]. Several cinnamic acid derivatives, including hydrocinnamic acid, 4-methylcinnamic acid, and *m*-coumaric acid, were also detected. Specifically, hydrocinnamic acid was found in cultures of strain D2, 4-methylcinnamic acid was found in in cultures of strain TK1, and *m*-coumaric acid was found exclusively in the co-culture of strain D2 with *B. cinerea* and *R. solani*. Notably, *m*-coumaric acid was absent from the control samples (Table 1). Table 2 contains an image showing the concentration of cinnamic acid in the D4 strain co-cultured with *Monilinia laxa*. Other compounds with documented antimicrobial effects include indole, 3-methylindole, and indole-3-carboxylic acid [36]. Importantly, these compounds also play roles in modulating plant immune responses to biotic and abiotic stresses [37]. In the present study, the production of indole was confirmed in co-cultures of *Metschnikowia pulcherrima* strains D2 and TK1 with phytopathogens, but not in the corresponding control samples. Indole-3-carboxylic acid was identified only in the co-culture of strain D2 with *M. laxa*, while 3-methylindole was detected in co-cultures of strain D2 with *B. cinerea*, *R. solani*, and *Alternaria alternata* (Table 1). These findings suggest that indole and its derivatives may be involved in plant defense mechanisms [37]. Lactic acid and oleic acid, both primary metabolites with antimicrobial activity, were also detected. Lactic acid, widely recognized for its industrial applications and antimicrobial effects [38], was found in the co-culture of strain D2 with *A. alternata*, as well as in both the control and co-culture samples of strain D4 (Table 1). Oleic acid, produced in all samples by strain TK1, has both antimicrobial activity and a regulatory role in plant immune responses [39,40]. Table 2 presents images showing the concentration gradients of lactic acid in strain D2 co-cultured with *A. alternata* and strain D4 with *M. laxa*. Pulcherriminic acid, a characteristic metabolite of *M. pulcherrima*, was also detected. As noted in the introduction, this compound chelates iron ions upon environmental release, forming pulcherrimin [10,23]. In this study, pulcherriminic acid was identified in the co-culture of strain TK1 with *A. alternata* (Table 1).

3,5-Dimethoxyphenol, another compound with antimicrobial activity [41], was detected in co-cultures of strain D2 (Table 1). Anisic acid (4-methoxybenzoic acid), an aromatic compound commonly found in anise, was identified in all the co-cultures of the tested strains. Yeasts are known to produce these aromatic compounds, and anisic acid has demonstrated antibacterial and antifungal activity [42] (Table 1). Azelaic acid was detected in the co-culture of strain D2 with *A. alternata* (Table 2), as well as in the control sample of strain TK1 (Table 1). This compound, which is naturally produced by plants in response to stress, can also be synthesized by certain microorganisms, including *Malassezia furfur* [43]. Perillic acid and syringic acid were detected in co-cultures of strains D4 and TK1, while phenylacetic acid was found in the co-cultures of all strains, except in the control samples. This pattern may reflect a yeast-mediated response to the presence of phytopathogens (Table 1). All these compounds exhibit antimicrobial activity [44,45,46]. Additionally, phenylacetic acid is a known plant hormone involved in developmental regulation. It is also produced by several microbial species, including *Enterobacter cloacae*, *Bacillus licheniformis*, and *Streptomyces humidus* [46].

#### 2.2.2. Untargeted Ultra-High-Performance Liquid Chromatography–Quadrupole Time-of-Flight Ultra-High Resolution Mass Spectrometry and Tandem Mass Spectrometry (UHPLC-QToF-UHRMS and MS/MS)

**Figure 2:**
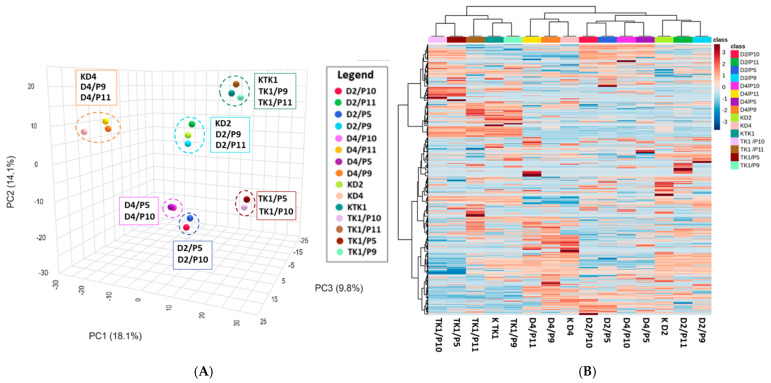
Statistical analysis of the entire MS data set. (**A**) Principal Component Analysis (PCA) score plot of metabolites from cultures of *M. pulcherrima* yeast D2, D4, and TK1 (controls: KD2, KD4, KTK11) and co-cultures of *M. pulcherrima* yeast strains D2, D4, and TK1 with phytopathogenic molds (P5—*Rhizoctonia solani*, P9—*Botrytis cinerea*, P10—*Monilia laxa*, P11—*Alternaria alternata*); (**B**) heatmap of metabolite abundance, distinguishing cultures of *M. pulcherrima* yeast strains D2, D4, and TK1, and co-cultures of *M. pulcherrima* yeast strains D2, D4, and TK1 with phytopathogenic molds (P5—*R. solani*, P9—*B. cinerea*, P10—*M. laxa*, P11—*A. alternata*). Red indicates higher relative abundance; blue indicates lower relative abundance. The color bar on the right represents the class labels for samples. Analysis performed via UHPLC-QToF-UHRMS.

A substantial number of chemical compounds (*N* = 344 to 2458) and metabolic pathways (*N* = 17 to 64) were identified during UHPLC-QToF-UHRMS analysis (Table 3). The numbers of compounds detected in the individual systems varied, indicating differences in the metabolic profiles of the various co-cultures. The numbers of metabolic pathways in the yeast monocultures ranged from 61 to 64, while in co-cultures with *B. cinerea* the number of metabolic pathways was lower, between 46 and 49 (Table 3). This may be due to the fact that in co-cultures, microorganisms competed for the environment and nutrients, which resulted in different metabolic responses. The numbers of differing metabolic pathways in yeast monocultures were generally lower than the numbers of differing pathways observed in co-cultures. This indicates that the presence and type of pathogens induce a distinct metabolic response in *M. pulcherrima* yeasts. Principal Component Analysis (PCA) allowed for the grouping of samples based on metabolite similarity. The principal components (PCA1, PCA2, PCA3) (Figure 2A) together account for 42% of the total variance in the data, which reflects the differences among the data sets. The plot shows six clusters, in which each *M. pulcherrima* yeast strain forms a separate group with either the control, *B. cinerea* and *A. alternata*, or *M. laxa* and *R. solani*. This may indicate differences between the strains as well as variations in metabolites depending on the presence and type of phytopathogen. The heat map (Figure 2B) illustrates the diversity of metabolic profiles among the samples. Additionally, it illustrates the expression levels of metabolites within each sample. PCA analysis suggests that the metabolic response of the yeasts varied depending on the co-culture system. Different yeast strains have distinct metabolic profiles, and the presence of phytopathogens induces a varied metabolic response. Similarities were observed for co-cultures with *B. cinerea* and *A. alternata*, as well as for co-cultures with *M. laxa* and *R. solani*.

Enrichment analysis enabled the comparison and classification of compounds and metabolic pathways. During the analysis, graphs were produced presenting the Enrichment overview, which showed the most important classes of compounds (Table 4). Detailed results are presented in the Supplementary Materials (Table S8).

The main differences between *M. pulcherrima* strains D2 and D4 grown alone and in co-cultures with phytopathogens (*R. solani*, *B. cinerea*, *M. laxa*, *A. alternata*) involved compound classes, such as carboxylic acids and derivatives, benzene derivatives, fatty acids, phenols, and organooxygen compounds. Key pathway differences included cinnamaldehydes, tetrahydroisoquinolines, and purine nucleosides. Strain TK1 showed notable differences in similar compound classes, with additional variation in indoles and derivatives. It produced more acidic compounds in the presence of the pathogens compared to D2 and D4 (Table 4).

Statistical analysis of the control samples (D2, D4, TK1) confirmed differences mainly in carboxylic acids, benzene derivatives, indoles, phenols, fatty acids, and flavonoids, with pathway differences in cinnamaldehydes, phenylpropanoic acids, and tetrahydroisoquinolines. In co-cultures, the most variable compound classes were fatty acids, phenols, benzene, and indole derivatives, organooxygen compounds, steroids, carboxylic acids, and flavonoids. Pathway differences were specific to each pathogen, with the most distinct changes observed in cinnamaldehydes, tetrahydroisoquinolines, phenylpropanoic acids, purine nucleosides, and ribonucleoside 3-phosphates (Table 4).

Throughout the LARAPPI/CI-MSI analysis, compounds such as indoles and cinnamaldehydes were detected, which is consistent with the presence of metabolic pathways associated with these compounds. In the case of phenols and flavonoids, this is particularly important, as these compounds exhibit anti-inflammatory and antimicrobial properties [47]. The presence of pathways and compound classes related to fatty acids is associated with the oleaginous nature of *M. pulcherrima* yeast [48]. The presence of benzene and carboxylic acid compound classes may be related to yeast metabolism; for example, the occurrence of benzene suggests an active aromatic compound degradation system [49].

During the metabolomic analysis, numerous compounds associated with primary metabolism were identified, including nucleosides, amino acids, and fatty acids, such as pinolenic acid (Table S9). As mentioned above, the presence of these metabolites in the *Metschnikowia pulcherrima* metabolome may be attributed to the oleaginous nature of this yeast species [50]. Metabolomic profiling also enabled the detection of antimicrobial compounds, many of which were previously localized using the LARAPPI/CI MSI method. These included anisic acid, azelaic acid, syringic acid, cinnamic acid and its derivatives, as well as indole and its derivatives, all of which were consistently detected across all samples (Table S9). In a study by Fernandez-San Millan et al. (2022), the authors conducted metabolomic analyses of yeast in both a monoculture and co-culture with *Botrytis cinerea*. One notable compound reported was catechol, which was also detected in every sample analyzed in this study [2]. Catechol possesses antimicrobial properties and is known to exert toxic effects on various microorganisms [50]. The presence of this common antimicrobial compound in all studies may indicate its metabolic importance for the *Metschnikowia pulcherrima* yeast. Another compound identified in all samples was salicylic acid, along with its derivatives. Salicylic acid is recognized for its broad-spectrum antimicrobial activity, particularly against fungal pathogens. It is widely applied in the management of postharvest diseases caused by *Penicillium expansum*, *B. cinerea*, *Fusarium oxysporum*, and *Rhizopus stolonifer* [51,52,53]. Humulene, a plant-derived compound detected in all samples, is known for its antimicrobial and antibiofilm properties [54]. Eucalyptol, another plant-origin compound with potent antimicrobial activity, was identified in the control sample of strain D4 [55]. A noteworthy compound identified in monocultures of strains D2 and D4 was dihydrojasmonic acid, a derivative of jasmonic acid. Jasmonic acid is a key phytohormone involved in plant stress responses and developmental processes [56]. Quinoline and its derivatives were detected in all analyzed samples. These compounds are known for their broad-spectrum antimicrobial activity, exhibiting potent effects against bacteria, such as *Escherichia coli* and *Staphylococcus aureus*, as well as antifungal effects against *Candida albicans* and *Aspergillus niger* [57]. Additionally, the control samples contained ricinoleic acid and monolaurin. Some glycosidic derivatives of ricinoleic acid have been reported to possess antibacterial activity, and ricinoleic acid-based polymers have demonstrated high efficacy against bacterial strains, such as *E. coli* [58]. Monolaurin, a derivative of lauric acid, is a well-documented antimicrobial agent, particularly effective against *S. aureus* [59].

*Metschnikowia pulcherrima* yeast can be used in wine production, and thanks to its properties enhances the organoleptic qualities of the final product. Many of the detected compounds, including eucalyptol, cinnamic acid, anisic acid, and perillic acid, are aromatic in nature and may influence the aroma of fermented products [34,42,43,55]. Not only are these properties potentially attractive to the fermented beverage industry, but they also enhance antimicrobial activity, as demonstrated in this study.

## 3. Materials and Methods

### 3.1. Microorganisms

#### 3.1.1. *Metschnikowia pulcherrima* Strains

Five strains of *M. pulcherrima* isolated from flowers and fruits were used. Strains D1 and D2 were isolated from apple fruits, strains D3 and D4 from raspberry fruits, and strain TK1 from strawberry flowers. Strains D1, D2, D3, and D4 were identified by sequencing the D1/D2 variable domains of the larger RNA subunit gene (LSU) [25], and the TK1 strain was identified by Maldi-TOF mass spectrometry, with a confidence score value in the range of 96.2–99.9% [5]. The yeasts were cultivated on YPG agar (yeast extract 1%, peptone 2%, glucose 2%, agar 1.5%) (BTL, Łódź, Poland) for 48 h at temp. 30 °C and stored at 4 °C. The suspensions of yeast cells were prepared in 0.85% NaCl (standardized according to 2 on the McFarland scale).

#### 3.1.2. Phytopathogen Strains

Eleven strains of phytopathogenic molds were used in this study. *Alternaria alternata*, *A. solani* Z 184, and *Fusarium oxysporum* Z 154 were obtained from the Collection of Pure Cultures of the Institute of Plant Breeding and Acclimatization of the National Research Institute (Radzikow, Poland). *Alternaria tenuissima* DSM 63360, *Colletotrichum coccodes* DSM 62126, *Fusarium sambucinum* DSM 62397, *Phoma exigua* DSM 62040, and *Rhizoctonia solani* DSM 22843 were obtained from Collection of Pure Cultures of the Leibniz Institute DSMZ (Germany). *Botrytis cinerea* and *Monilia laxa* were obtained from the Pathogen Bank Institute of Plant Protection National Research Institute (Poznań, Poland). *Venturia inaequalis* was obtained from the Plant Protection Department, National Research Institute of Horticulture (Skierniewice, Poland). All phytopathogenic strains were cultivated on Potato Dextrose Agar (PDA) [3.9%] (Merck, Darmstadt, Germany) for 10–14 days at temp. 25 °C and stored at 4 °C. The suspensions of fungal strains were prepared by collecting spores from the surface of the pure culture on PDA and resuspended in 0.85% NaCl for a final concentration of 10^4^ CFU/mL (counting was performed in a Thoma chamber).

### 3.2. Antimicrobial Activity Assessment

The agar-well diffusion method was used to assess the antimicrobial activity of the yeast strains against phytopathogens. YPG liquid medium (BTL, Lodz, Poland) was prepared in volumes of 50 mL and inoculated with a 1 mL of suspension yeast cells. Cultures were carried out for 48 h at temp. 25 °C with shaking (130 rpm, Heidolph Unimax 1010 shaker). PDA plates were inoculated with 100 µL of mold suspension. Next, wells were cut out using a sterile cork borer with a diameter of 10 mm, and 150 µL of yeast cell suspension or yeast culture was transferred into the wells. After inoculation, the plates were incubated at temp. 25 °C for 10 days. The diameters of pathogen growth-inhibition zones were measured [mm], excluding the well width. The results are expressed as the average of 3 independent repetitions for each combination *M. pulcherrima* strain and phytopathogen strain with a standard deviation value. Additionally, negative controls were performed using medium without yeast (no growth inhibition was observed in the control samples).

### 3.3. Co-Cultivation of M. Pulcherrima and Phytopathogens

The co-culturing of yeast *M. pulcherrima* and phytopathogens was conducted on malt extract [5%] (MEB) (BTL, Łódź, Poland). Based on the antimicrobial activity test, yeast–phytopathogen systems were selected for co-cultivation: *M. pulcherrima* yeast strains D2, D4, and TK1, and *A. alternata*, *R. solani*, *B. cinerea*, and *M. laxa*. For this purpose, MEB medium in a volume of 50 mL was inoculated with a 1 mL of suspension yeast cells and shaken at speed 180 rpm (Heidolph unimax 1010 shaker) at a temp. range of 22–25 °C for 24 h. Then, the yeast cultures were inoculated with 1 mL of mold spore suspension (10^4^ CFU/mL) and the co-cultures were incubated for 14 days. Control monocultures of the phytopathogens and yeasts were also performed under the same conditions. The numbers of yeast cells were counted using the microscopic method in a Thoma counting chamber. Cell-free supernatants were obtained by centrifugation (9000 rpm, 10 min, room temperature), filtered through 0.22 µm syringe filters (GenoPlast Biotech S.A., Rokocin, Poland), and the sterile supernatants were submitted to metabolomic analysis using the UHPLC-QToF-UHRMS method. The samples for this study using the LARAPPI/CI MSI method consisted of PDA plates with surface-inoculated phytopathogens (100 µL spore suspension) and a disc onto which the yeast culture was applied (15 µL of a 48 h yeast culture prepared as described in Material and Methods 3.1. Co-cultures on both liquid and solid media were performed in a single replicate.

### 3.4. Metabolomic Analysis

#### 3.4.1. Laser Ablation Remote Atmospheric Pressure Photoionization/Chemical Ionization Mass Spectrometry Imaging (LARAPPI/CI MSI)

For metabolomic imaging using the LARAPPI/CI MSI method, agar plates were prepared with a co-culture of yeasts and phytopathogens. *M. pulcherrima* yeast strains D2, D4, and TK1, and *Alternaria alternata*, *Rhizoctonia solani* DSM 22843, *Botrytis cinerea*, and *Monilia laxa* were selected. Specific setups exhibited large inhibition zones of phytopathogen growth.

The LARAPPI/CI MSI system, first described in a recent publication [28], is based on an airtight chamber pressurized with nitrogen gas to produce a nitrogen stream of 10 L/min. The sample was placed on a 50 × 50 mm sample stage, with a Peltier cooling plate that sustained the sample at −18 °C. The temperature-controlled sample stage was mounted on a motorized high-speed XY-stage. The pulsed beam from the OPO laser (2.93 µm, 7 ns, 20 Hz, 3.5 mJ/pulse) entered the sample chamber through a sapphire window. Then, the beam was expanded to 3.75 -times and was redirected toward the sample stage by a gold mirror. The beam then passed through a diffractive optical element forming a square-shape top-hat beam. It was focused onto the sample surface by a 50 mm focal-length aspherical ZnSe lens. The optical assembly and the camera with a lens and distance sensor were mounted on aluminum rails and were in a fixed configuration; the only moving parts were the XYZ stages. During imaging, the laser focal point remained fixed in space, while the sample was moved. A specially designed gas funnel also functioned as a focusing assembly and was connected to a 6/4 mm (O.D/I.D.) PTFE tube. The overpressure in the chamber drove a 10 L/min nitrogen gas flow through the tube. The laser ablation plumes were entrained into the gas and transported to the modified ion source (Bruker VIP HESI in the APCI configuration) of a Bruker Impact II mass spectrometer. The ion source also had a VUV source (Hamamatsu L12542) mounted axially to the MS sampling cone inside. An HPLC pump (Agilent G1312A) provided a steady flow of a solvent mixture (1% toluene in methanol; 200 μL/min) to the APCI needle [28]. The settings of the ion source were as follows: APCI nebulizer, end plate offset at 600 V, capillary at 1000 V, corona at 6000 nA, nebulizer at 3.5 bar, dry gas at 0.2 L/min, dry temperature at 250 °C, probe gas temperature at 350 °C, probe gas at 4 L/min, and exhaust turned on. MS^1^ experiments were performed with the following settings: frequency at 3 Hz and scan range of *m*/*z* 47–1300.

The 2D-MSI experiment was performed with different resolutions, numbers of voxels, and ablation region sizes for each co-culture, due to the nature of the samples. Exact test parameters are presented in the Supplementary Materials (Table S1). Each voxel in the 2D-MSI experiment was exposed to the laser for 1 s, at a laser pulse repetition rate of 20 Hz. The delays between pixels were 1000 ms. Between pixels, the sample stage moved at a speed of 50 mm/s. The time delay between lines was 5 s. The objects in individual Petri dishes were cut with a blade (average size of 25 × 25 mm) and placed on a stainless-steel plate (0.8 mm thickness). The plates were then placed on an ablation table inside the chamber and frozen (−18 °C).

#### 3.4.2. Untargeted Ultra-High-Performance Liquid Chromatography–Quadrupole Time-of-Flight Ultra-High Resolution Mass Spectrometry and Tandem Mass Spectrometry (UHPLC-QToF-UHRMS)

Metabolomic profiling was carried out for supernatants of each co-culture of yeast and phytopathogens, as well as the control samples (yeast culture only or pure medium). Solid-phase extraction (SPE) was performed to extract and concentrate metabolites from the supernatants. After conditioning an SPE C18 column with 1 mL of methanol and equilibrating it by washing twice with 1 mL of water, 4 mL of supernatant was gradually introduced onto the column. The column was then washed with 2 mL of water and eluted with 1 mL of methanol. The samples were dried for 24 h in a speedvac-type apparatus under the following conditions: 1300 rpm and 0.1 mbar vacuum. The pellet formed after drying was suspended in 120 µL of methanol and vortexed briefly. To facilitate dissolution, the samples were sonicated for a few seconds and then centrifuged for 5 min at 12,000 rpm using a mySPIN™ 12 Mini Centrifuge (Thermo Fisher Scientific, Waltham, MA, USA). Supernatants in a volume of 80 µL were transferred to glass inserts compatible with standard HPLC vials. UHPLC-QToF-UHRMS analysis was performed using a Bruker Elute UHPLC system operated with Hystar 3.3 software, coupled to a high-resolution (60,000+) Bruker Impact II ESI QTOF mass spectrometer (Bruker Daltonics GmbH, Bremen, Germany). Data acquisition and processing were conducted using MetaboScape version 2022b and DataAnalysis 4.2 version 2022b (Bruker Daltonics GmbH). AutoMSMS measurements were performed using a Bruker UHPLC Intensity Solo Column (C18, dimensions 100 × 2.1 mm, 2 μm particles). Eluents A (water with 0.1% HCOOH) and B (acetonitrile with 0.1% HCOOH) were applied. For the measurements, the following percentages were used: 0 min and 2 min—99% A; 17 min—1% A; 20 min—1% A; and 20.1 min, 22 min, 30 min—99% A. The flow rate was maintained at 0.25 μLmin^−1^ from 0 min to 20 min and at 0.35 μLmin^−1^ from 20.1 min to 30 min. The samples in the autosampler were kept at 4 °C. The column was held at 40 °C. The exit of the column was connected to the ESI source. The injection volume on the column was 5 μL.

The mode *m*/*z* range used for autoMSMS was 50–1200. Collision-Induced Dissociation was applied with the following settings: absolute area threshold: 5000 counts; active exclusion: 2 spectra; release after 0.3 min; isolation mass: for *m*/*z* = 100, width was 4, for *m*/*z* = 300 width was 5, for *m*/*z* = 500 width was 6, for *m*/*z* = 1000 width was 8; and the collision energy value was 30 eV. Internal calibration on 10 mM sodium formate (water:isopropanol 1:1 *v*/*v*) ions was carried out automatically in Metaboscape ver. 2022b with the use of a syringe pump at an infusion flow rate of 1.5 μL/min, using the high-precision calibration (HPC) mode. Untargeted annotations were performed in Metaboscape ver. 2022b with a criterion of mass deviation (Δ*m*/*z*) under 3 ppm and an mSigma value under 100 s as the maximum acceptable deviations of the mass of the compound and the isotopic pattern, respectively. All the molecular formulas were obtained using the Smart Formula tool and the C, H, N, O, P, S, Cl, Br, I, and F elements. MSMS spectra were automatically matched against MSMS libraries: Bruker HMDB 2.0 library, MassBank of North America (MoNA) library (Mass Bank of North America, 2024) and NIST ver. 2023 MSMS library (Mass Spectrometry Data Center, 2022). Detailed annotation parameters for each compound, including accurate mass deviation (Δ*m*/*z*), isotope pattern score (mSigma), retention time match (if available), and MS/MS spectral matching score (0–1000 scale), are provided in the Supplementary Tables S9, allowing the independent assessment of annotation confidence.

### 3.5. Statistical Analysis

The mean numbers of yeasts cells in the co-cultures with molds, as well as the diameters of the pathogen growth-inhibition zones, were compared using a one-way analysis of variance (ANOVA) at a significance level of 0.05. When a statistical difference was detected (*p* < 0.05), the means were compared using Tukey’s post hoc procedure at a significance level of 0.05. Statistical analysis was carried out using R Project for Statistical Computing 4.4.3 (R Core Team, Auckland, New Zealand).

MetaboAnalyst 6.0 was used for analysis of metabolomics data. The data were log-transformed and auto-scaled. Fold change analysis was conducted to identify metabolites with substantial differences between groups, using thresholds of FC > 2 and FC < 0.5. Heatmaps were generated based on normalized and auto-scaled data, using the Euclidean distance and Ward’s clustering method. Pathway enrichment analysis was performed using the Kyoto Encyclopedia of Genes and Genomes (KEGG) database, with reference organisms relevant to fungal metabolism, including *Pichia kudriavzevii*, *Cryptococcus neoformans*, *Aspergillus clavatus*, *Coccidioides immitis*, and *Aspergillus niger*. The aim was to identify significantly enriched metabolic pathways associated with differential metabolites. Enrichment analysis of the main compound classes was performed based on the Small Molecule Pathway Database (SMPDB) to identify the predominant chemical categories contributing to the observed group differences. Statistical comparisons between more than two groups were performed using a non-parametric one-way analysis of variance (Kruskal–Wallis test) at a significance level of 0.05.

## 4. Conclusions

This research constitutes the first investigation of yeast–phytopathogen co-cultures with *Rhizoctonia solani*, *Botrytis cinerea*, *Monilinia laxa*, and *Alternaria alternata*, employing the LARAPPI/CI MSI methodology, which facilitated the identification of compounds potentially involved in the inhibition of mold growth. Metabolomic analysis yielded comprehensive insights into the metabolic profiles of the yeast strains and revealed the influence of the phytopathogens on the yeast cellular response. The current study supports the hypotheses that the *Metschnikowia pulcherrima* yeast tested in interactions with various phytopathogenic fungi changed its metabolic profile depending on the fungal strain. In addition to producing typical antimicrobial compounds, the yeast synthesized metabolites with antimicrobial properties specific to particular fungal strains. Furthermore, a broad spectrum of previously unreported metabolites with antifungal activity was identified in *M. pulcherrima* cultures. The findings of this study confirm the antimicrobial activity of the tested *M. pulcherrima* isolates and underscore their broad metabolic potential.

The application of UHPLC-QToF-UHRMS enabled the detection of antimicrobial metabolites (for example, indole and its derivatives, lactic acid, pulcherriminic acid, salicylic acid), which may contribute to the observed antagonistic effects, highlighting the potential of *M. pulcherrima* in biocontrol strategies. Aromatic compounds with antimicrobial activity were also detected, including eucalyptol, cinnamic acid, anisic acid, and perillic acid. These compounds also exhibit antimicrobial activity, which may be beneficial in the development of biopreparates.

This study provides a strong basis for further research on plants under in vivo conditions. Future research aimed at developing a biopreparation based on *M. pulcherrima* represents a scientific challenge. To achieve this goal, it will be necessary to optimize culture conditions, including the growth medium. The next step should involve developing the formulation of the biopreparation, including the selection of the form in which the yeast will be delivered, the protective substances, and the storage conditions. Additionally, future studies should focus on transcriptomic and metabolomic analyses on apple plant (leaves, fruits) treated with *M. pulcherrima* yeast and subsequently infected by phytopathogenic fungi. Analyses of gene expression and active metabolites involved in the plant–pathogen–yeast interactions will indicate specific bioprotective mechanisms of *M. pulcherrima* yeast, as well as plant defense mechanisms following phytopathogenic invasion. These analyses will extend the knowledge of the pathogenesis and bioprotective mechanisms of unconventional yeasts. The continuation of this research holds great promise and could lead to the development of a practical and eco-friendly product.

## Figures and Tables

**Table 1:** Identification via LARAPPI/CI-MSI analysis of chemical compounds synthesized by *Metschnikowia pulcherrima* yeast strains D2, D4, and TK1 in control samples and during co-cultivation with molds (*Botrytis cinerea*, *Rhizoctonia solani*, *Alternaria alternata*, *Monilia laxa*).

No	Compounds	*M. pulcherrima* D2			*M. pulcherrima* D4			*M. pulcherrima* TK1				
		Control	*B. cinerea*	*R. solani*	Control	*B. cinerea*	*R. solani*	Control	*B. cinerea*	*R. solani*	*A. alternata*	*M. laxa*
1	2-Ketobutyric acid ^a,b^	−	−/−	+/−	+	+/−	+/−	+	+/+	+/+	+/−	+/+
2	Serine ^a,b^	−	+/+	+/−	+	+/+	+/+	+	−/+	−/−	+/−	−/−
3	Proline ^a,b,c,d^	−	−/−	+/−	+	+/+	+/+	−	+/−	−/−	+/+	+/−
4	Indole ^a,b,c,d^	−	+/−	+/−	n.d.	n.d.	n.d.	−	+/−	−	+/−	+/−
5	3-Hydroxyisovaleric acid ^a,b^	+	+/−	+/−	n.d.	n.d.	n.d.	n.d.	n.d.	n.d.	n.d.	n.d.
6	2-Hydroxy-2-methylbutyric acid ^a,b^	n.d.	n.d.	n.d.	+	+/−	+/−	+	+/−	−/−	+/−	+/−
7	Threonine ^a,b^	+	+/+	+/−	+	+/+	+/+	−	+/−	−	+/−	+/+
8	Histidine ^a,b^	n.d.	n.d.	n.d.	−	+/−	+/−	n.d.	n.d.	n.d.	n.d.	n.d.
9	Phenylalanine ^a,b^	n.d.	n.d.	n.d.	+	+/+	+/+	−	+/+	−/−	+/−	+/−
10	Perillic acid ^a,b,c^	n.d.	n.d.	n.d.	+	+/+	+/−	+	+/−	+/−	+/−	+/−
11	*o*-Tyrosine ^a,b,c^	n.d.	n.d.	n.d.	−	+/−	+/−	n.d.	n.d.	n.d.	n.d.	n.d.
12	Pipecolic acid ^a,b^	−	−/−	+/−	+	+/+	+/−	n.d.	n.d.	n.d.	n.d.	n.d.
13	3-Methylindole ^a,c,d^	−	+/+	+/−	n.d.	n.d.	n.d.	n.d.	n.d.	n.d.	n.d.	n.d.
14	Glutamic acid ^a^	+	+/+	+/−	+	+/+	+/+	+	+/−	+/−	+/−	+/−
15	2-Hydroxycaproic acid ^a,b,c^	+	+/−	+/−	+	+/+	+/+	−	+/+	+/−	+/−	+/−
16	Malic acid ^a,b^	−	−/−	+/−	n.d.	n.d.	n.d.	n.d.	n.d.	n.d.	n.d.	n.d.
17	Lactic acid ^a^	n.d.	n.d.	n.d.	+	+/−	+/−	n.d.	n.d.	n.d.	n.d.	n.d.
18	3,4-Dimethylbenzaldehyde ^a,c,d^	+	+/−	+/−	n.d.	n.d.	n.d.	+	−/−	−/+	+/−	+/−
19	Phenylacetic acid ^a,b,c^	−	+/−	+/−	−	+/+	+/+	−	+/−	+/+	−/−	**+/−**
20	Syringic acid ^a,b^	n.d.	n.d.	n.d.	+	+/−	+/−	+	+/−	+/−	+/−	+/−
21	4-Hydroxyquinoline ^a,c,d^	−	−/+	+/+	n.d.	n.d.	n.d.	n.d.	n.d.	n.d.	n.d.	n.d.
22	Cinnamic acid ^a,d^	+	+/+	+/−	n.d.	n.d.	n.d.	n.d.	n.d.	n.d.	n.d.	n.d.
23	Hydrocinnamic acid ^a,b,d^	+	+/−	+/−	n.d.	n.d.	n.d.	n.d.	n.d.	n.d.	n.d.	n.d.
24	4-Methylcinnamic acid ^a,d^	n.d.	n.d.	n.d.	n.d.	n.d.	n.d.	−	−/−	−/+	−/−	−/+
25	*p*-Anisic acid ^a,b^	+	+/−	+/−	+	+/−	+/−	+	+/−	+/−	+/−	+/−
26	3,5-Dimethoxyphenol ^a,b,c^	+	+/−	+/−	n.d.	n.d.	n.d.	n.d.	n.d.	n.d.	n.d.	n.d.
27	*m*-Coumaric acid ^a,b,c^	−	+/+	+/−	n.d.	n.d.	n.d.	n.d.	n.d.	n.d.	n.d.	n.d.
28	4-Pyridoxic acid ^a,b^	n.d.	n.d.	n.d.	n.d.	n.d.	n.d.	−	−/+	−/−	−/−	−/+
29	5-Hydroxytryptophan ^a,b^	n.d.	n.d.	n.d.	n.d.	n.d.	n.d.	+	+/−	+/−	+/−	+/−
30	Pulcherriminic acid ^a^	n.d.	n.d.	n.d.	n.d.	n.d.	n.d.	−	−/−	−/−	+/−	−/−
31	Oleic acid ^a,c^	n.d.	n.d.	n.d.	n.d.	n.d.	n.d.	−	+/−	+/−	+/−	+/−

^a^ Annotated based on high precursor mass accuracy obtained from UHPLC-UHRMS; ^b^ annotated based on retention time match obtained from UHPLC-UHRMS; ^c^ annotated based on isotopic pattern fit (mSigma) obtained from UHPLC-UHRMS; ^d^ annotated based on MS/MS fragment spectra matching obtained by tandem mass spectrometry obtained from UHPLC-UHRMS; control—*M. pulcherrima yeast* D2, D4, and TK1 monocultures; (+/–) present/absent in yeast/mold colony; n.d.—not detected.

**Table 2:** Selected antimicrobial compounds produced by *Metschnikowia pulcherrima* yeast strains D2 and D4 in contact with phytopathogenic molds *Alternaria alternata* and *Monilia laxa* on PDA medium (LARAPPI/CI MSI analysis).

Name of Chemical Compound	Ion Image	Name of Chemical Compound	Ion Image
*Metschnikowia pulcherrima* yeast D2 (center) and *Alternaria alternata* culture grown on PDA medium—control pre-ablation	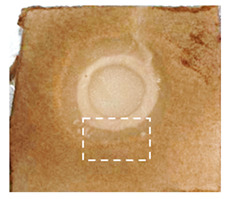	*Metschnikowia pulcherrima* yeast D4 (center) and *Monilia laxa* culture grown on PDA medium—control pre-ablation	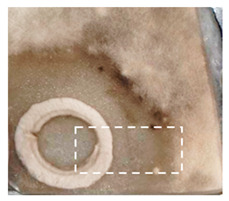
Acetic acid	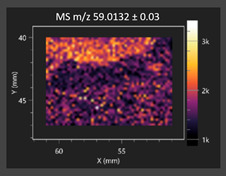	Acetic acid	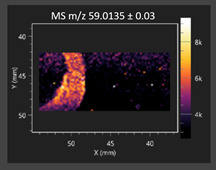
Pyruvaldehyde	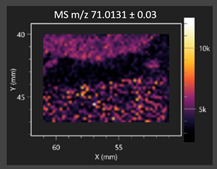	Pyruvaldehyde	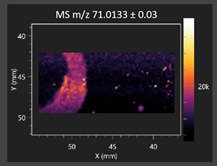
Lactic acid	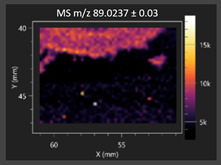	Lactic acid	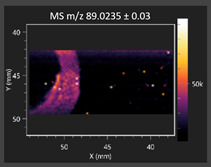
3-Phenyllactic acid	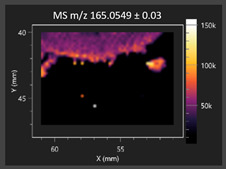	Succinic acid semialdehyde	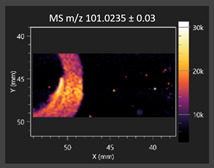
Azelaic acid	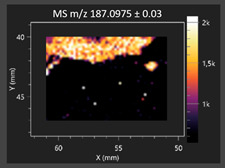	Cinnamic acid	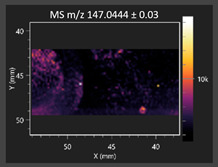

**Table 3:** Comparison analysis via UHPLC-QToF-UHRMS of metabolic pathways and chemical compounds identified in cultures of *M. pulcherrima* yeast (strains: D2, D4, TK1) and co-cultures of yeasts with phytopathogenic molds (P5—*R. solani*, P9—*B. cinerea*, P10—*M. laxa*, P11—*A. alternata*).

Samples Compared	Number of Metabolic Pathways Identified *	Number of Chemical Compounds Identified ^#^
M.p D2	63	1002
M.p D4	64	2458
M.p TK1	61	713
M.p D2/P9	46	344 ^#^
M.p D4/P9	49	855 ^#^
M.p TK1/P9	47	830 ^#^
M.p D2 vs. M.p D4 vs. M.p TK1	17–19 *	336 ^#^
M.p D2/P5 vs. M.p D4/P5 vs. M.p TK1/P5	23–26 *	477 ^#^
M.p D2/P9 vs. M.p D4/P9 vs. M.p TK1/P9	26–30 *	511 ^#^
M.p D2/P10 vs. M.p D4/P10 vs. M.p TK1/P10	24–25 *	479 ^#^
M.p D2/P11 vs. M.p D4/P11 vs. M.p TK1/P11	24–28 *	519 ^#^

* Ranges refer to differences due to the microorganism databases used for comparison: *Pichia kudriavzevii*, *Cryptococcus neoformans*, and *Aspergillus clavatus*; # in the case of the numbers of metabolites in the co-cultures, the number of metabolites takes into account only those that differed from the control yeast culture.

**Table 4:** Pathway enrichment analysis results for cultures of *M. pulcherrima* yeast D2, D4, and TK1, and co-cultures yeast with phytopathogenic molds. Analysis performed via UHPLC-QToF-UHRMS.

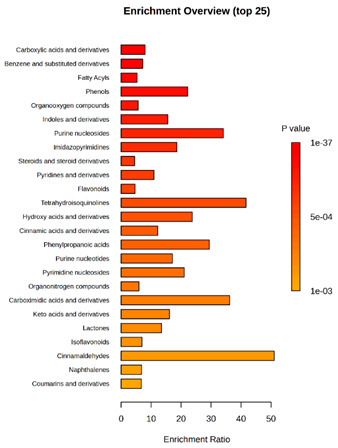 (KD2 vs. D2/P5, D2/P9, D2/P10, D2/P11)	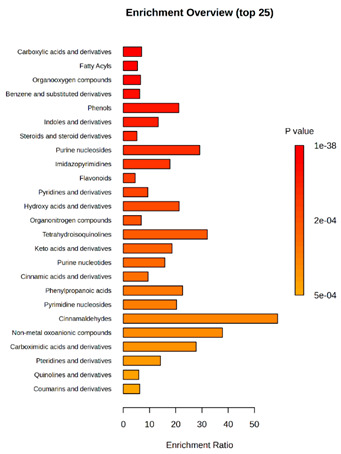 KD4 vs. D4/P5, D4/P9, D4/P10, D4/P11)
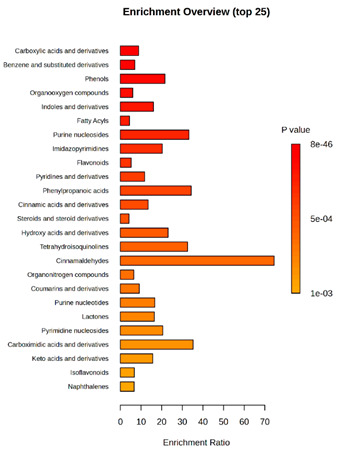 (KTK1 vs. TK1/P5, TK1/P9, TK1/P10, TK1/P11)	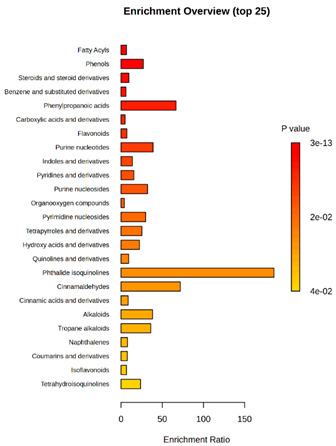 D2 vs. D4 vs. TK1
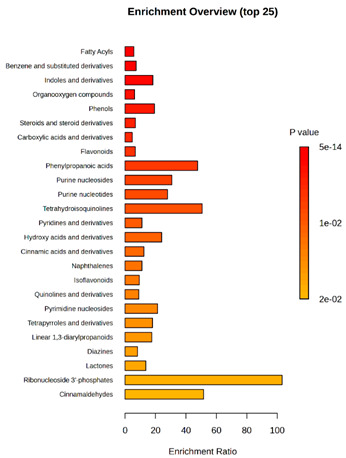 D2/P5 vs. D4/P5 vs. TK1/P5	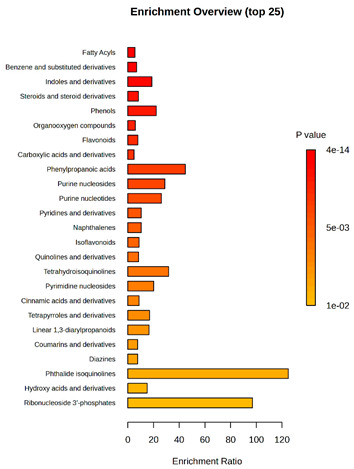 D2/P9 vs. D4/P9 vs. TK1/P9
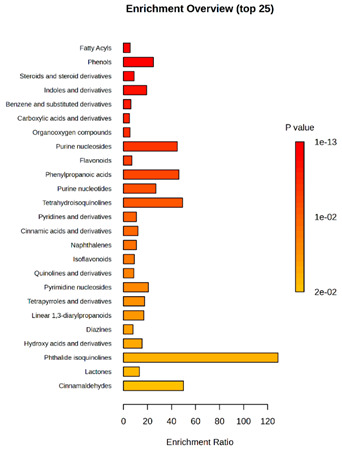 D2/P10 vs. D4/P10 vs. TK1/P10	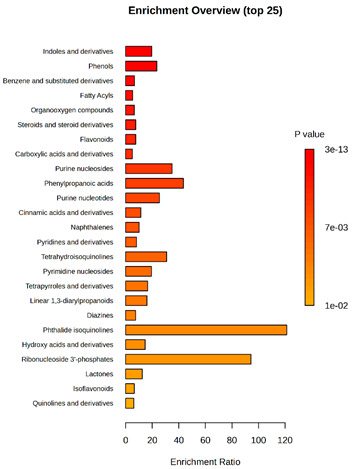 D2/P11 vs. D4/P11 vs. TK1/P11

K—yeast controls: KD2, KD4, and KTK1; molds: P5—*R. solani*, P9—*B. cinerea*, P10—*M. laxa*, and P11—*A. alternata*. Bar plot of enriched chemical classes showing the enrichment ratio for each class. The color scale indicates statistical significance (*p*-value), with red indicating higher significance.

## Data Availability

All research data can be found in the Supplementary Materials.

## Supplementary Materials

The following supporting information can be downloaded at: https://www.mdpi.com/article/10.3390/molecules30153268/s1, Table S1: MSI parameters for studied objects; Table S2: Growth inhibition zones of phytopathogens depending on the yeast strain *M. pulcherrima*; Table S3: Number of *M. pulcherrima* cells in co-culture with molds; Table S4: Identification of chemical compounds synthesized by *Metschnikowia pulcherrima* yeast D2, D4, and TK1 in control samples and during co cultivation with phytopathogenic moulds (*Botrytis cinerea*, *Rhizoctonia solani*, *Alternaria alternata*, *Monilia laxa*) performed via LARAPPI/CI analysis.; Table S5: Identification of chemical compounds produced by *Metschnikowia pulcherrima* yeast, strain D2 in control sample and during co-cultivation with phytopathogenic moulds (*Rhizoctonia solani* and *Botrytis cinerea*) performed via LARAPPI/CI analysis; Table S6: Identification of chemical compounds produced by *Metschnikowia pulcherrima* yeast, strain D4 in control sample and during co-cultivation with phytopathogenic moulds (*Rhizoctonia solani* and *Botrytis cinerea*) performed via LARAPPI/CI analysis; Table S7: Identification of chemical compounds produced by *Metschnikowia pulcherrima* yeast, strain TK1 in control sample and during co-cultivation with phytopathogenic moulds (*Rhizoctonia solani*, *Botrytis cinerea*, *Alternaria alternata* and *Moinilia laxa*) performed via LARAPPI/CI analysis; Table S8: Metabolic pathways detected during UHPLC-QToF-UHRMS analysis; Table S9: metabolites detected during UHPLC-QToF-UHRMS analysis
