# Supplementary material for: Metabolomic Insights into the Antimicrobial Effects of Metschnikowia Yeast on Phytopathogens
Source: Molecules. 2025 Aug 4;30(15):3268. doi: 10.3390/molecules30153268 (PMC12348177; doi:10.3390/molecules30153268)
Supplement: Supplementary file 1 [file molecules-30-03268-s001.zip › supplementary materials tables S1-S7.pdf]

Table S1. MSI parameters for studied objects

Sample name	Resolution [µm]	MSI region dimensions [mm]	Number of voxels (X x Y)
**control yeast strain TK1 sample**	230	X: 6.80<br>Y: 5.80	35 x 30
**yeast strain TK1 with *Botrytis cinerea***	350	X: 17.50<br>Y: 11.20	51 x 33
**yeast strain TK1 with *Rhizoctonia solani***	230	X: 11.50<br>Y: 6.90	51 x 31
**yeast strain TK1 with *Alternaria alternata***	200	X: 12.00<br>Y: 6.00	61 x 31
**yeast strain TK1 with *Monilia laxa***	250	X: 14.00<br>Y: 7.00	57 x 29
**control yeast strain D2 sample**	250	X: 10.00<br>Y: 6.50	41 x 27
**yeast strain D2 with *Rhizoctonia solani***	260	X: 13.00<br>Y: 8.32	51 x 33
**yeast strain D2 with *Botyris cinerea***	250	X: 13.00<br>Y: 6.75	53 x 28
**yeast strain D2 with *Monilia laxa***	190	X: 14.82<br>Y: 4.94	79 x 27
**yeast strain D2 with *Alternaria alternata***	190	X: 9.88<br>Y: 6.84	53 x 37
**control yeast strain D4 sample**	230	X: 8.26<br>Y: 6.44	37 x 29
**yeast strain D4 with *Botrytis cinerea***	260	X: 13.00<br>Y: 8.32	51 x 33
**yeast strain D4 with *Rhizoctonia solani***	230	X: 10.58<br>Y: 7.36	47 x 33
**yeast strain D4 with *Monilia laxa***	200	X: 16.40<br>Y: 7.2	83 x 37
**yeast strain D4 with *Alternaria alternata***	150	X: 7.80<br>Y: 4.80	53 x 33

Table S2. Growth inhibition zones of phytopathogens depending on the yeast strain *M. pulcherrima*

No.	Phytopathogen	Growth Inhibition Zone [mm] Mean ± SD									
		M. pulcherrima D1		*M. pulcherrima* D2		*M. pulcherrima* D3		*M. pulcherrima* D4		*M. pulcherrima* TK1	
		culture	cells suspension	culture	cells suspension	culture	cells suspension	culture	cells suspension	culture	cells suspension
1	*Alternaria alternata**	5±0	5±0	10±0	15±7,0	10±0	27,5±10,6	11±1,4	12,5±10,6	11,5±2,12	10,5±0,7
2	*Alternaria solani*	6±0^{abc}	17,5±3,5^{a}	5±1,4^{bc}	nd^{c}	5,5±2,1^{abc}	6±1,4^{abc}	14±8,4^{ab}	4±1,4^{bc}	14,1,4^{ab}	5±0^{bc}
3	*Alternaria tenuissima**	17,5±10,6	16±5,7	17,5±3,5	22,5±3,5	14,5±0,7	15±0	12,5±3,5	10±8,4	15±7,1	13,5±2,1
4	*Botrytis cinerea*	12,5±10,6^{c}	20±0^{bc}	47,5±3,5^{a}	52,5±10,6^{a}	22,5±3,5^{bc}	32,5±3,5^{abc}	37,5±10,6^{ab}	30±0^{abc}	42,5±3,5^{ab}	35±0^{abc}
5	*Colletotrichum coccodes*	nd^{b}	nd^{b}	12,5±10,6^{ab}	22,5±10,6^{a}	3±1,4^{b}	7±0^{ab}	6,5±0,7^{ab}	7,5±0,7^{ab}	2±0^{b}	2±0^{b}
6	*Fusarium oxysporum*	nd^{c}	nd^{c}	22,5±3,5^{ab}	25±7,0^{a}	22,5±3,5^{ab}	11,5±0,7^{bc}	20±0^{ab}	12±0^{b}	18,5±2,1^{ab}	18±2,8^{ab}
7	*Fusarium sambucinum**	nd	nd	16,5±12,0	17,5±10,6	16,5±2,12	19±8,5	12±1,4	18±2,8	20±0	15±0
8	*Monilia laxa**	20±0	19,5±3,5	17,5±3,5	30±0	13,5±2,12	15±0	30±14,14	40±10,6	32,5±10,6	27,5±17,7
9	*Phoma exigua*	15±7,0^{def}	17,5±3,5^{cdef}	25±7,0^{bcd}	30±0^{bc}	3±0^{f}	20±0^{cde}	17,5±3,5^{cdef}	10±0^{ef}	37,5±3,5^{ab}	45±0^{a}
10	*Rhizoctonia solani*	22,5±3,5^{ab}	45±0^{ab}	40±3,5^{ab}	37,5±7,0^{ab}	40±7,0^{ab}	45±0^{ab}	40±0^{ab}	50±0^{a}	20±14,1^{b}	20±14,1^{b}
11	*Venturia inaequalis*	nd^{d}	nd^{d}	22,5±3,5^{ab}	15±7,0^{abc}	22,5±3,5^{ab}	10±0^{bcd}	12±7,0^{abcd}	25±0^{a}	15±0^{abc}	7,5±3,5^{cd}

nd – inhbition non detected, * the variations in the results were not statistically significant, statistically different samples are marked with different letters within the same rows (a,b,c...) (Tukey's post hoc test at a significance level of 0.05).

Table S3. Number of *M. pulcherrima* cells in co-culture with molds

No	Co-culture with phytopathogenic moulds	*M. pulcherrima* strains [cells/ml]		
		D2	D4	TK1
1	control sample*	**M:$6{,}0\times10^{9}$ a**<br>SD: $5{,}7\times10^{7}$	M: $2{,}0\times10^{9}$ ab<br>SD: $3{,}6\times10^{6}$	M:$4{,}0\times10^{9}$ ab<br>SD: $1{,}3\times10^{9}$
2	*Rhizoctonia solani*	M:3.1×10^{9} b<br>SD: $6{,}4\times10^{8}$	M:1.4×10^{9} abc<br>SD:$1{,}3\times10^{8}$	**M:4.8×10^{9} a**<br>SD: $5{,}6\times10^{8}$
3	*Botrytis cinerea*	M:8.9×10^{8} cd<br>SD: $2{,}6\times10^{6}$	M:1.2×10^{9} abc<br>SD: $2{,}3\times10^{7}$	M:**5.1×10^{9} a**<br>SD: $6{,}0\times10^{7}$
4	*Monilia laxa*	M:1.8×10^{9} c<br>SD: $4{,}3\times10^{8}$	**M:2.4×10^{9} a**<br>SD:$4{,}4\times10^{7}$	M:2.2×10^{9} b<br>SD: $1{,}7\times10^{8}$
5	*Alternaria alternata*	M:1.7×10^{9} c<br>SD: $6{,}1\times10^{8}$	M:4.4×10^{8} bc<br>SD:$6{,}9\times10^{8}$	**M:4.4×10^{9} a**<br>SD: $1{,}4\times10^{9}$

* - without molds, only *M. pulcherrima* yeast, M- mean, SD – standard deviation, sample number N=3; **Bold -** the most intensive growth in coculture with a given phytopathogen;, statistically different samples are marked with different letters within the same column (a,b,c) (Tukey's post hoc test at a significance level of 0.05)

Table S4. Identification of chemical compounds synthesized by *Metschnikowia pulcherrima* yeast D2, D4, and TK1 in control samples and during co-cultivation with phytopathogenic moulds (*Botrytis cinerea, Rhizoctonia solani, Alternaria alternata, Monilia laxa*) performed via LARAPPI/CI analysis.

control - *M. pulcherrima yeast* D2, D4, and TK1 colony without phytopathogenic moulds; (+/-) present/absent in yeast/mould colony; nd – not detected

Compounds	*M.pulcherrima* D2					*M.pulcherrima* D4					*M.pulcherrima* TK1				
	**Control**	***B. cinerea***	***R. solani***	***A. alternata***	***M. laxa***	**Control**	***B. cinerea***	***R. solani***	***A. alternata***	***M. laxa***	**Control**	***B. cinerea***	***R. solani***	***A. alternata***	***M. laxa***
Acetic acid	nd	nd	nd	+/-	nd	nd	nd	nd	nd	+/-	nd	nd	nd	nd	nd
N-Acetyl-L-alanine	nd	nd	nd	nd	-/+	nd	nd	nd	nd	+/+	nd	nd	nd	nd	nd
1-Acetyl beta carboline	nd	nd	nd	-/+	nd	nd	nd	nd	nd	nd	nd	nd	nd	nd	nd
Acetyl-L-carnitine	nd	nd	nd	nd	-/+	nd	nd	nd	nd	nd	nd	nd	nd	nd	nd
β-N-Acetylglucosamine	nd	nd	nd	nd	-/+	nd	nd	nd	-/+	nd	nd	nd	nd	nd	nd
N-acetyl DL-methionine	nd	nd	nd	nd	+/-	nd	nd	nd	nd	nd	nd	nd	nd	nd	nd
Acetylphosphate	nd	nd	nd	-/+	nd	nd	nd	nd	nd	nd	nd	nd	nd	nd	nd
N-Acetyltryptophan	nd	nd	nd	+/-	nd	nd	nd	nd	nd	nd	nd	nd	nd	nd	nd
Trans-Aconitic acid	nd	nd	nd	nd	-/+	nd	nd	nd	nd	nd	nd	nd	nd	nd	nd
Adenine	nd	nd	nd	nd	nd	nd	nd	nd	-/+	nd	nd	nd	nd	nd	nd
Adipic acid	nd	nd	nd	nd	+/+	nd	nd	nd	+/+	nd	nd	nd	nd	nd	nd
Alanine	nd	nd	nd	nd	nd	nd	nd	nd	nd	+/-	nd	nd	nd	nd	nd
D-Alpha-aminobutyric acid	nd	nd	nd	+/-	nd	nd	nd	nd	nd	nd	nd	nd	nd	nd	nd
p-Aminobenzoic acid	nd	nd	nd	nd	-/+	nd	nd	nd	nd	nd	nd	nd	nd	nd	nd
3-Aminoisobutanoic acid	nd	nd	nd	nd	nd	nd	nd	nd	nd	nd	+/-	nd	nd	nd	nd
5-Aminopentanoic acid	nd	nd	nd	+/-	+/+	nd	nd	nd	nd	nd	nd	nd	nd	nd	nd
p-Anisic acid	+	+/-	+/-	nd	nd	+	+/-	+/-	nd	nd	+	+/-	+/-	+/-	+/-
Arabitol	nd	nd	nd	nd	+/-	nd	nd	nd	+/-	nd	+/-	nd	nd	nd	nd
Arginine	nd	nd	nd	+/-	nd	nd	nd	nd	nd	nd	nd	nd	nd	nd	nd
Aspartic acid	nd	nd	nd	+/+	+/-	nd	nd	nd	+/-	+/-	nd	nd	nd	nd	nd
Azelaic acid	nd	nd	nd	+/-	nd	nd	nd	nd	nd	nd	+/-	nd	nd	nd	nd
Betaine	nd	nd	nd	nd	+/+	nd	nd	nd	+/-	nd	+/-	nd	nd	nd	nd
Biotin	nd	nd	nd	nd	nd	nd	nd	nd	nd	nd	+/-	nd	nd	nd	nd
Canavanine	nd	nd	nd	-/+	nd	nd	nd	nd	nd	nd	nd	nd	nd	nd	nd
2-Carbamoylpyridine-3-carboxylic acid	nd	nd	nd	-/+	nd	nd	nd	nd	nd	nd	nd	nd	nd	nd	nd
5-Carboline	nd	nd	nd	nd	-/+	nd	nd	nd	nd	nd	nd	nd	nd	nd	nd
Cinnamic acid	+	+/+	+/-	nd	nd	nd	nd	nd	nd	+/+	nd	nd	nd	nd	nd
Citric acid	nd	nd	nd	+/+	nd	nd	nd	nd	nd	nd	nd	nd	nd	nd	nd
m-Coumaric acid	nd	+/+	+/-	-/+	-/+	nd	nd	nd	nd	nd	nd	nd	nd	nd	nd
m-Cresol	nd	nd	nd	-/+	nd	nd	nd	nd	nd	nd	nd	nd	nd	nd	nd
p-Cresol sulfate	nd	nd	nd	+/-	nd	nd	nd	nd	nd	nd	+/-	nd	nd	nd	nd
Cytosine	nd	nd	nd	-/+	-/+	nd	nd	nd	nd	nd	nd	nd	nd	nd	nd
Creatinine	nd	nd	nd	-/+	nd	nd	nd	nd	nd	nd	nd	nd	nd	nd	nd
1-Cyclobutyl-4-piperidinamine	nd	nd	nd	+/-	nd	nd	nd	nd	nd	nd	nd	nd	nd	nd	nd
Cyclo-prolylglycine	nd	nd	nd	-/+	+/-	nd	nd	nd	nd	nd	nd	nd	nd	nd	nd

Deoxyguanosine	nd	nd	nd	-/+	nd	nd	nd	nd	nd	nd	nd	nd	nd	nd	nd
2,4-diacetylphloroglucinol	nd	nd	nd	nd	-/+	nd	nd	nd	nd	nd	nd	nd	nd	nd	nd
Diaminopimelic acid	nd	nd	nd	nd	-/+	nd	nd	nd	nd	nd	-/+	nd	nd	nd	nd
3,4-Dihydroxymandelic acid	nd	nd	nd	nd	-/+	nd	nd	nd	-/+	nd	nd	nd	nd	nd	nd
2,3-Dihydroxbenzoic acid	nd	nd	nd	-/+	nd	nd	nd	nd	nd	nd	nd	nd	nd	nd	nd
2,6-Dihydroxy-4-methylbenzoic acid	nd	nd	nd	nd	-/+	nd	nd	nd	nd	nd	nd	nd	nd	nd	nd
3,4-Dimethylbenzaldehyde	+	+/-	+/-	-/+	+/-	nd	nd	nd	nd	nd	+	-/-	-/+	+/-	+/-
3,5 Dimethylbenzene 1,2-diamine	nd	nd	nd	nd	nd	nd	nd	nd	nd	nd	+/-	nd	nd	nd	nd
3,5-Dimethoxyphenol	+	+/-	+/-	nd		nd	nd	nd	nd	nd	nd	nd	nd	nd	nd
N-[5-(2,5-Dioxopyrrolidin-1-yl)pentyl]-N-hydroxyacetamide	nd	nd	nd	nd	+/-	nd	nd	nd	nd	nd	nd	nd	nd	nd	nd
5-Dodecenoic acid	nd	nd	nd	nd	+/-	nd	nd	nd	+/-	nd	nd	nd	nd	nd	nd
Ergothioneine	nd	nd	nd	nd	-/+	nd	nd	nd	nd	nd	nd	nd	nd	nd	nd
Erythrose	nd	nd	nd	+/+	nd	nd	nd	nd	nd	nd	nd	nd	nd	nd	nd
4-Ethylbenzoic acid	nd	nd	nd	+/-	nd	nd	nd	nd	nd	nd	+/-	nd	nd	nd	nd
Ethylmalonic acid	nd	nd	nd	+/+	nd	nd	nd	nd	nd	nd	nd	nd	nd	nd	nd
N-Formyl-L-methionine	nd	nd	nd	-/+	nd	nd	nd	nd	nd	nd	nd	nd	nd	nd	nd
D-Fructose	nd	nd	nd	nd	-/+	nd	nd	nd	nd	nd	nd	nd	nd	nd	nd
Fructoselysine	nd	nd	nd	+/-	nd	nd	nd	nd	nd	nd	+/-	nd	nd	nd	nd
N-Fructosyl isoleucine	nd	nd	nd	nd	nd	nd	nd	nd	nd	nd	+/+	nd	nd	nd	nd
L-Fucose	nd	nd	nd	nd	-/+	nd	nd	nd	nd	nd	nd	nd	nd	nd	nd
Fumaric acid	nd	nd	nd	-/+	nd	nd	nd	nd	nd	nd	nd	nd	nd	nd	nd
Galactitol	nd	nd	nd	nd	-/+	nd	nd	nd	nd	nd	nd	nd	nd	nd	nd
Gluconolactone	nd	nd	nd	nd	-/+	nd	nd	nd	nd	nd	nd	nd	nd	nd	nd
Glutamic acid	+	+/+	+/-	nd	+/+	+	+/+	+/+	+/+	nd	+	+/-	+/-	+/-	+/-
Glutamine	nd	nd	nd	nd	nd	nd	nd	nd	nd	nd	+/-	nd	nd	nd	nd
Glutathione	nd	nd	nd	nd	+/-	nd	nd	nd	nd	nd	+/-	nd	nd	nd	nd
Glycerophosphocholine	nd	nd	nd	nd	nd	nd	nd	nd	nd	nd	+/-	nd	nd	nd	nd
Guaifenesin	nd	nd	nd	nd	+/-	nd	nd	nd	nd	nd	nd	nd	nd	nd	nd
Hippuric acid	nd	nd	nd	-/+	nd	nd	nd	nd	-/+	nd	nd	nd	nd	nd	nd
Histidine	nd	nd	nd	+/-	+/+	-	+/-	+/-	nd	+/-	nd	nd	nd	nd	nd
3-oxo-C6-homoserine lactone	nd	nd	nd	nd	nd	nd	nd	nd	+/-	nd	nd	nd	nd	nd	nd
Hydrocinnamic acid	+	+/-	+/-	nd	-/+	nd	nd	nd	nd	nd	nd	nd	nd	nd	nd
2-Hydroxyadenine	nd	nd	nd	-/+	nd	nd	nd	nd	nd	nd	nd	nd	nd	nd	nd

3-Hydroxyanthranilic acid	nd	nd	nd	nd	+/-	nd	nd	nd	nd	nd	nd	nd	nd	nd	nd
4-Hydroxybutanoic acid lactone	nd	nd	nd	-/+		nd	nd	nd	nd	nd	nd	nd	nd	nd	nd
2-Hydroxybenzene-1,3-dicarboxylic acid	nd	nd	nd	nd	-/+	nd	nd	nd	nd	nd	nd	nd	nd	nd	nd
2-Hydroxycaproic acid	+	+/-	+/-	+/+	+/-	+	+/+	+/+	+/+	+/-	nd	+/+	+/-	+/-	+/-
Hydroxyphenyllactic acid	nd	nd	nd	-/+	nd	nd	nd	nd	nd	nd	nd	nd	nd	nd	nd
Hydroxyindoleacetic acid	nd	nd	nd	nd	+/-	nd	nd	nd	-/+			nd	nd	nd	nd
5-Hydroxy-2-(hydroxymethyl) pyridine	nd	nd	nd	-/+	nd	nd	nd	nd	nd	nd	nd	nd	nd	nd	nd
3-Hydroxyisovaleric acid	+	+/-	+/-	nd	nd	nd	nd	nd	nd	nd	nd	nd	nd	nd	nd
2-Hydroxy-2-methylbutyric acid	nd	nd	nd	nd	nd	+	+/-	+/-	nd	nd	+	+/-	nd	+/-	+/-
5-Hydroxymethyl-2-furancarboxylic acid	nd	nd	nd	-/+	-/+	nd	nd	nd	nd	nd	nd	nd	nd	nd	nd
5-Hydroxy-L-tryptophan	nd	nd	nd	nd	-/+	nd	nd	nd	nd	nd	+	+/-	+/-	+/-	+/-
4-Hydroxyquinoline	nd	-/+	+/+	nd	nd	nd	nd	nd	nd	nd	nd	nd	nd	nd	nd
Hypoxanthine	nd	nd	nd	-/+	nd	nd	nd	nd	nd	nd	nd	nd	nd	nd	nd
Hypotaurine	nd	nd	nd	nd	+/-	nd	nd	nd	nd	nd	nd	nd	nd	nd	nd
Imidazoleacetic acid	nd	nd	nd	-/+	-/+	nd	nd	nd	-/+	nd	nd	nd	nd	nd	nd
Indole	nd	+/-	+/-	nd	nd	nd	nd	nd	nd	nd	nd	+/-	nd	+/-	+/-
Indoline	nd	nd	nd	+/+	+/+	nd	nd	nd	+/-	nd	nd	nd	nd	nd	nd
Indole-3-carboxylic acid	nd	nd	nd	nd	+/-	nd	nd	nd	nd	nd	nd	nd	nd	nd	nd
3-Indoleacrylic acid; AIF; CE30; MS2Dec	nd	nd	nd	nd	-/+	nd	nd	nd	nd	nd	nd	nd	nd	nd	nd
2-Ketobutyric acid	nd	nd	+/-	nd	nd	+	+/-	+/-	nd	nd	+	+/+	+/+	+/-	+/+
Kojic acid	nd	nd	nd	nd	-/+	nd	nd	nd	nd	nd	nd	nd	nd	nd	nd
Kynurenic acid	nd	nd	nd	nd	+/-	nd	nd	nd	nd	nd	nd	nd	nd	nd	nd
Lactic acid	nd	nd	nd	+/-	nd	+	+/-	+/-	nd	+/-	nd	nd	nd	nd	nd
Levoglucosan	nd	nd	nd	nd	-/+	nd	nd	nd	nd	nd	nd	nd	nd	nd	nd
Malic acid	nd	nd	+/-	-/+	-/+	nd	nd	nd	-/+	-/+	nd	nd	nd	nd	nd
D-Maltose	nd	nd	nd	nd	+/-	nd	nd	nd	nd	nd	nd	nd	nd	nd	nd
Mannitol	nd	nd	nd	nd	-/+	nd	nd	nd	nd	nd	nd	nd	nd	nd	nd
Methionine	nd	nd	nd	nd	nd	nd	nd	nd	nd	nd	+/-	nd	nd	nd	nd
4-Methoxycoumarin	nd	nd	nd	-/+	nd	nd	nd	nd	-/+	nd	nd	nd	nd	nd	nd
3-Methoxytyramine	nd	nd	nd	+/-	nd	nd	nd	nd	+/-	nd	+/-	nd	nd	nd	nd
4-Methylacetophenone	nd	nd	nd	+/+	nd	nd	nd	nd	nd	nd	+/-	nd	nd	nd	nd

4-methylbenzene-carboximidamide	nd	nd	nd	+/-	nd	nd	nd	nd	nd	nd	nd	nd	nd	nd	nd
4-Methylcinnamic acid	nd	nd	nd	nd	nd	nd	nd	nd	nd	nd	nd	nd	-/+	nd	-/+
3-Methylindole	nd	+/+	+/-	+/+	nd	nd	nd	nd	nd	nd	nd	nd	nd	nd	nd
2-Methyl-3-ketovaleric acid	nd	nd	nd	nd	-/+	nd	nd	nd	nd	nd	nd	nd	nd	nd	nd
3-[5-(2-Methylpropyl)-3,6-dioxo piperazin-2-yl]propanoic acid	nd	nd	nd	nd	+/-	nd	nd	nd	nd	nd	nd	nd	nd	nd	nd
1-Methyl-1H-pyrido[2,3-b]indole	nd	nd	nd	nd	-/+	nd	nd	nd	nd	nd	nd	nd	nd	nd	nd
(E)-3,4-(Methylenedioxy) cinnamaldehyde	nd	nd	nd	nd	-/+	nd	nd	nd	nd	nd	nd	nd	nd	nd	nd
Methyl 2-(2-hydroxypro panoylamino)-3-methylbutanoate	nd	nd	nd	nd	-/+	nd	nd	nd	nd	nd	nd	nd	nd	nd	nd
3-Methyl-1-(2,4,6-trihydroxyphenyl)butan-1-one	nd	nd	nd	nd	-/+	nd	nd	nd	nd	nd	nd	nd	nd	nd	nd
2-Napthylamine	nd	nd	nd	-/+	nd	nd	nd	nd	-/+	nd	nd	nd	nd	nd	nd
Nicotinic acid	nd	nd	nd	-/+	nd	nd	nd	nd	-/+	nd	nd	nd	nd	nd	nd
4-Nitrosodiphenylamine	nd	nd	nd	nd	nd	nd	nd	nd	+/-	nd	nd	nd	nd	nd	nd
Norleucine	nd	nd	nd	+/-	+/-	nd	nd	nd	+/-	nd	+/+	nd	nd	nd	nd
p-Octopamine	nd	nd	nd	nd	+/-	nd	nd	nd	nd	nd	+/-	nd	nd	nd	nd
Oleic acid	nd	nd	nd	nd	nd	nd	nd	nd	nd	nd	nd	+/-	+/-	+/-	+/-
Ornithine	nd	nd	nd	+/-	nd	nd	nd	nd	nd	nd	nd	nd	nd	nd	nd
2-Oxoarginine	nd	nd	nd	nd	-/+	nd	nd	nd	nd	nd	-/+	nd	nd	nd	nd
Oxypurinol	nd	nd	nd	nd	nd	nd	nd	nd	nd	nd	+/-	nd	nd	nd	nd
Pantothenic acid	nd	nd	nd	+/+	+/+	nd	nd	nd	+/+	nd	+/-	nd	nd	nd	nd
Paraxanthine	nd	nd	nd	nd	-/+	nd	nd	nd	nd	nd	nd	nd	nd	nd	nd
Perillic acid	nd	nd	nd	nd	nd	+	+/+	+/-	nd	nd	+	+/-	+/-	+/-	+/-
Phenylalanine	nd	nd	nd	nd	+/+	+	+/+	+/+	nd	nd	nd	+/+	nd	+/-	+/-
Phenylacetaldehyde	nd	nd	nd	nd	nd	nd	nd	nd	nd	+/-	nd	nd	nd	nd	nd
Phenylacetic acid	nd	+/-	+/-	nd	nd	nd	+/+	+/+	nd	nd	nd	+/-	+/+	nd	+/-
Phenethylacetate	nd	nd	nd	nd	-/+	nd	nd	nd	nd	nd	nd	nd	nd	nd	nd
L-3-Phenyllactic acid	nd	nd	nd	+/-	+/+	nd	nd	nd	nd	nd	+/-	nd	nd	nd	nd
Phosphorylcholine	nd	nd	nd	nd	-/+	nd	nd	nd	nd	nd	-/+	nd	nd	nd	nd
Pipecolic acid	nd	nd	+/-	nd	nd	+	+/+	+/-	nd	nd	nd	nd	nd	nd	nd
Proline	nd	nd	+/-	-/+	+/+	+	+/+	+/+	nd	nd	nd	+/-	nd	+/+	+/-
Pulcherriminic acid	nd	nd	nd	nd	nd	nd	nd	nd	nd	nd	nd	nd	nd	+/-	nd

4-Pyridoxic acid	nd	nd	nd	nd	nd	nd	nd	nd	nd	nd	nd	-/+	nd	nd	-/+
Pyroglutamic acid	nd	nd	nd	nd	-/+	nd	nd	nd	nd	nd	nd	nd	nd	nd	nd
Pyromucic acid	nd	nd	nd	-/+	-/+	nd	nd	nd	-/+	nd	nd	nd	nd	nd	nd
Pyridoxamine	nd	nd	nd	+/+	+/+	nd	nd	nd	nd	nd	+/-	nd	nd	nd	nd
Pyridoxin	nd	nd	nd	nd	nd	nd	nd	nd	-/+	nd	nd	nd	nd	nd	nd
PyroGlu-Pro	nd	nd	nd	+/-	-/+	nd	nd	nd	nd	nd	nd	nd	nd	nd	nd
PyroGlu-Val	nd	nd	nd	nd	-/+	nd	nd	nd	nd	nd	nd	nd	nd	nd	nd
Pyrrolidonecarboxylic acid	nd	nd	nd	-/+	-/+	nd	nd	nd	-/+	nd	+/-	nd	nd	nd	nd
Pyruvaldehyde	nd	nd	nd	+/+	nd	nd	nd	nd	nd	+/-	nd	nd	nd	nd	nd
Rhamnose	nd	nd	nd	nd	-/+	nd	nd	nd	nd	nd	nd	nd	nd	nd	nd
Ribitol	nd	nd	nd	+/-	+/-	nd	nd	nd	nd	nd	nd	nd	nd	nd	nd
Serine	nd	+/+	+/-	nd	+/+	+	+/+	+/+	nd	+/+	+	-/+	nd	+/-	nd
Sorbose	nd	nd	nd	-/+	-/+	nd	nd	nd	-/+	nd	+/-	nd	nd	nd	nd
Sorbitol	nd	nd	nd	nd	-/+	nd	nd	nd	nd	nd	nd	nd	nd	nd	nd
Succinic acid semialdehyde	nd	nd	nd	+/-	nd	nd	nd	nd	nd	+/-	nd	nd	nd	nd	nd
Succinylacetone	nd	nd	nd	+/-	+/-	nd	nd	nd	nd	nd	+/-	nd	nd	nd	nd
Syringic acid	nd	nd	nd	nd	nd	+	+/-	+/-	nd	nd	+	+/-	+/-	+/-	+/-
Terrein	nd	nd	nd	nd	nd	nd	nd	nd	nd	nd	+/-	nd	nd	nd	nd
5,6,7,8-Tetrahydro-2-naphthalenecarboxylic acid	nd	nd	nd	nd	-/+	nd	nd	nd	nd	nd	nd	nd	nd	nd	nd
Threonine	+	+/+	+/-	nd	nd	+	+/+	+/+	nd	+/+	nd	+/-	nd	+/-	+/+
Trehalose	nd	nd	nd	-/+	+/-	nd	nd	nd	nd	nd	+/-	nd	nd	nd	nd
Triethyl phosphate	nd	nd	nd	nd	nd	nd	nd	nd	nd	nd	-/+	nd	nd	nd	nd
Tryptophanol	nd	nd	nd	nd	+/+	nd	nd	nd	nd	nd	+/-	nd	nd	nd	nd
Thymidine	nd	nd	nd	nd	+/-	nd	nd	nd	nd	nd	nd	nd	nd	nd	nd
Thymine	nd	nd	nd	nd	-/+	nd	nd	nd	nd	nd	nd	nd	nd	nd	nd
Tyrosine	nd	nd	nd	nd	+/+	nd	+/-	+/-	+/+	nd	nd	nd	nd	nd	nd
Uracil	nd	nd	nd	nd	-/+	nd	nd	nd	nd	nd	nd	nd	nd	nd	nd
Vanillylmandelic acid	nd	nd	nd	+/-	nd	nd	nd	nd	nd	nd	nd	nd	nd	nd	nd
Quinic acid	nd	nd	nd	+/-	+/-	nd	nd	nd	+/-	nd	+/-	nd	nd	nd	nd

Table S5. Identification of chemical compounds produced by *Metschnikowia pulcherrima* yeast, strain **D2** in control sample and during co-cultivation with phytopathogenic moulds (*Rhizoctonia solani* and *Botrytis cinerea)* performed via LARAPPI/CI analysis

<table>
<tr><th>Compound</th><th>yeast strain D2 (left) agar gel (right)</th><th>yeast strain D2 (left) Rhizoctonia solani (right)</th><th>yeast strain D2 (left) Botrytis cinerea (right)</th></tr>
<tr><td></td><td>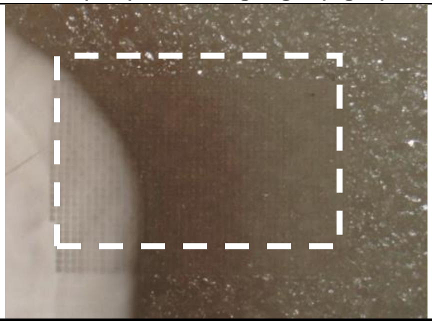</td><td>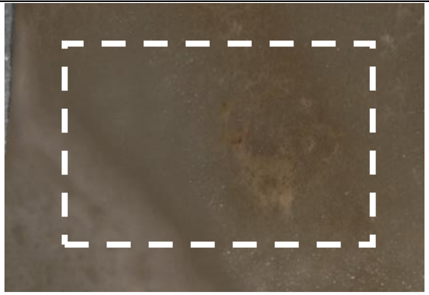</td><td>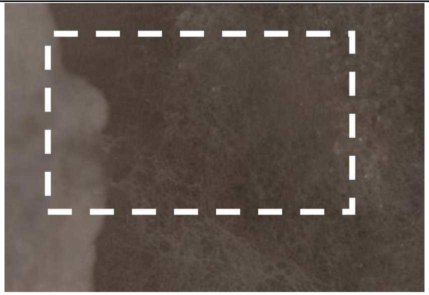</td></tr>
<tr><td>2-Ketobutyric acid</td><td>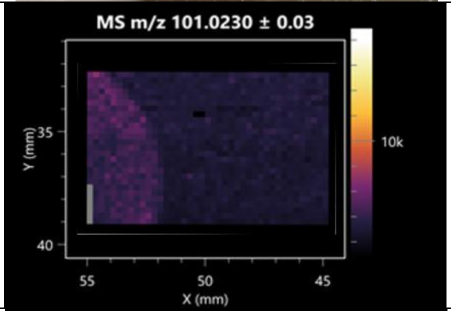
</td><td>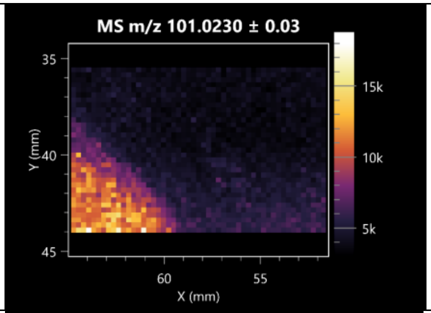
</td><td>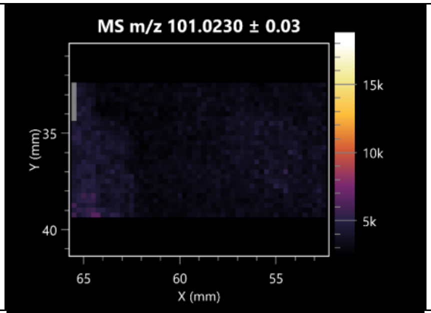
</td></tr>
<tr><td>Serine</td><td>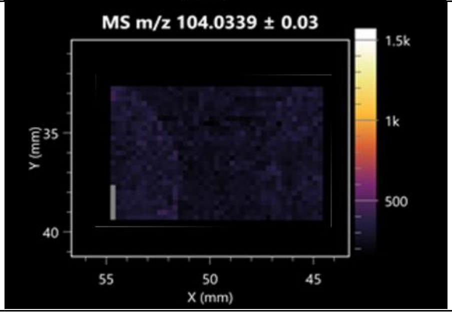
</td><td>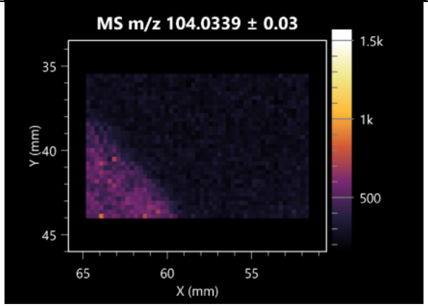
</td><td>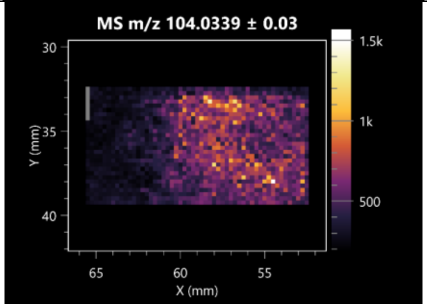
</td></tr>
</table>

Proline	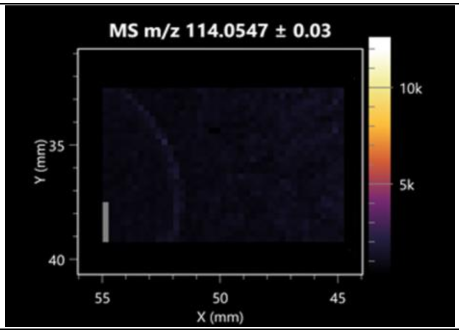	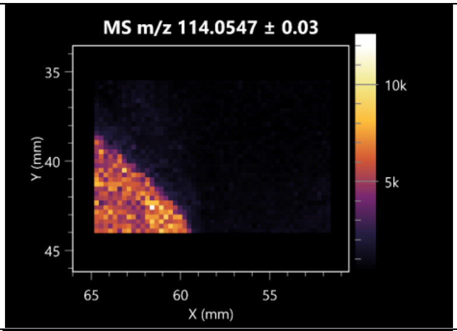	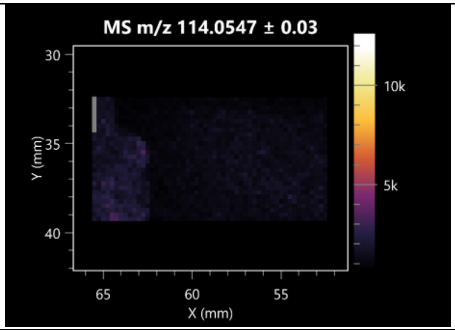
Indole	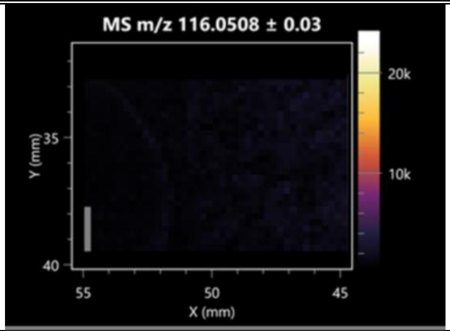	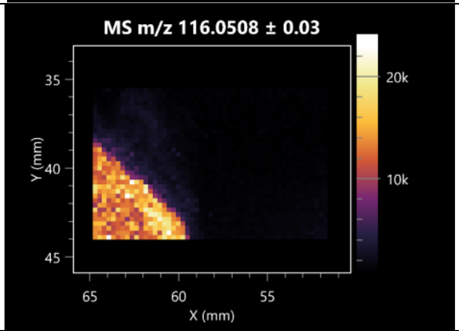	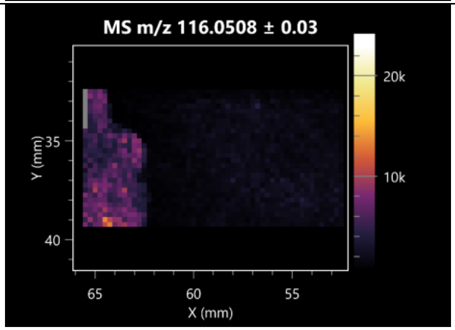
3-Hydroxyisovaleric acid	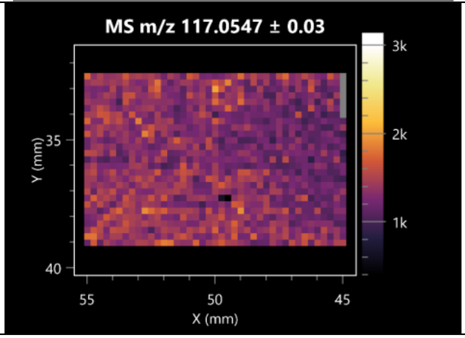	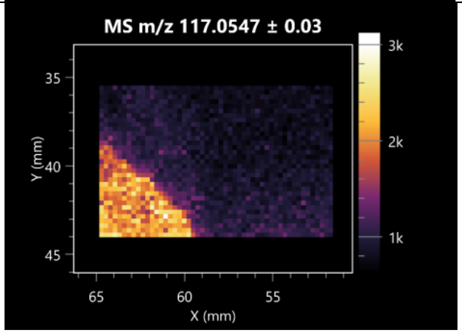	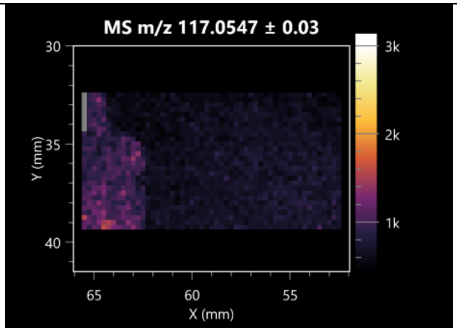

<table>
<tr><td>Threonine</td><td>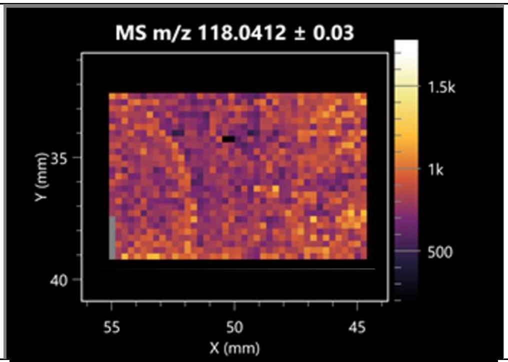
</td><td>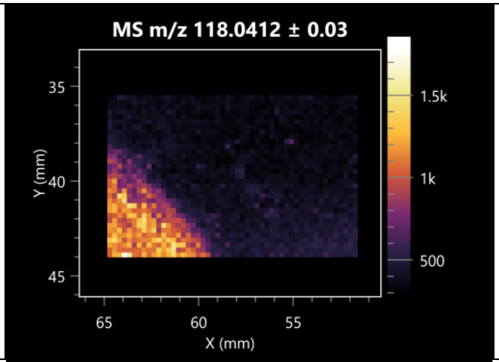
</td><td>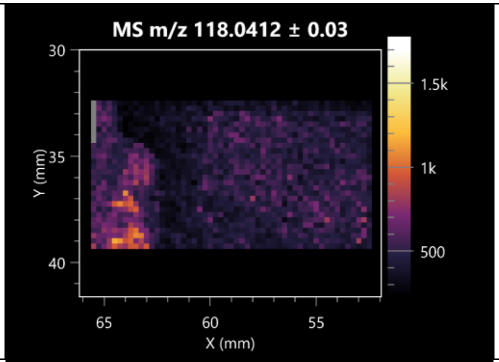
</td></tr>
<tr><td>Pipecolic acid</td><td>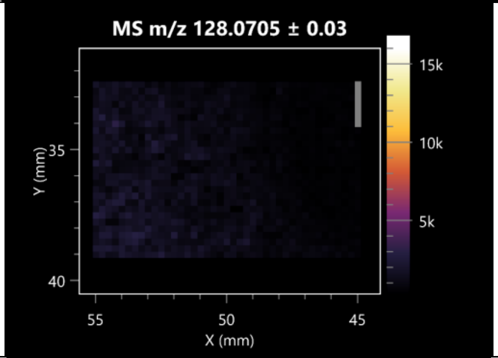
</td><td>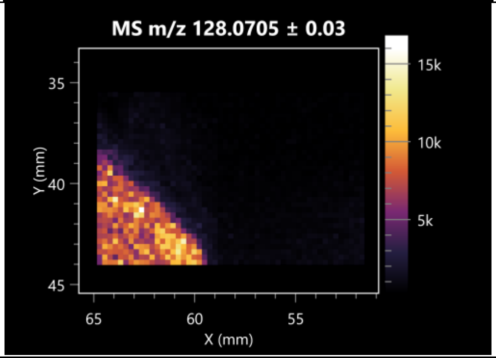
</td><td>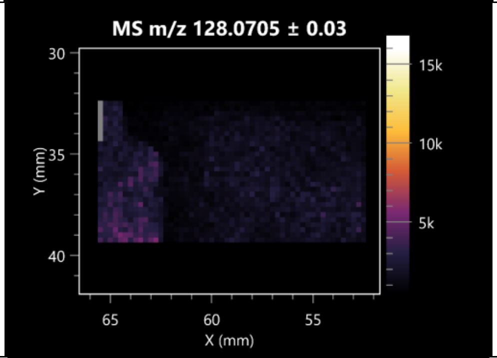
</td></tr>
<tr><td>3-Methylindole</td><td>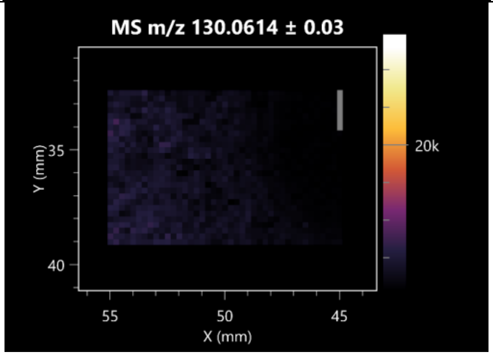
</td><td>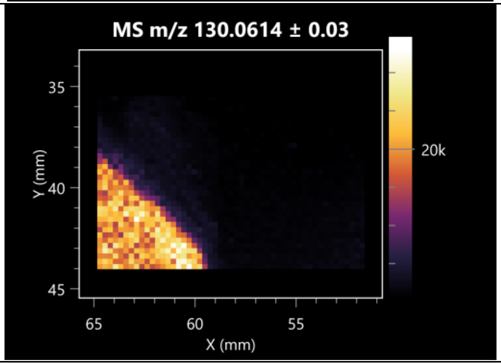
</td><td>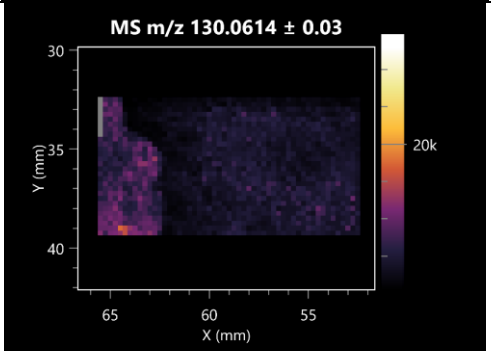
</td></tr>
</table>

Glutaric acid	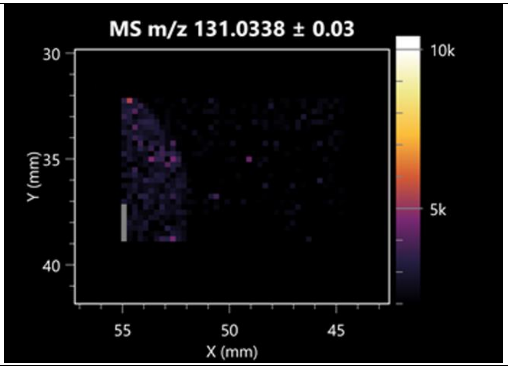	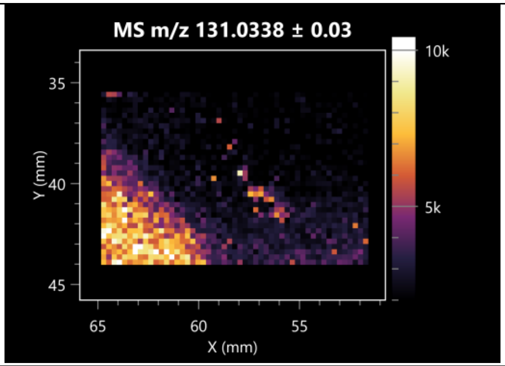	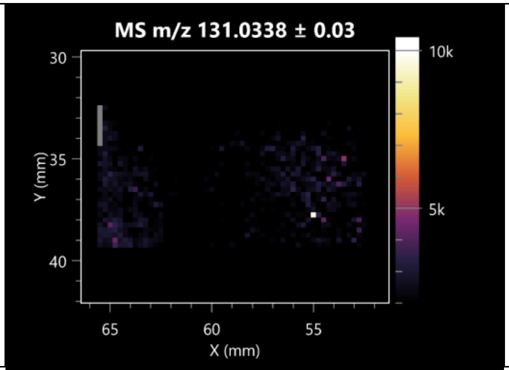
2-Hydroxycaproic acid	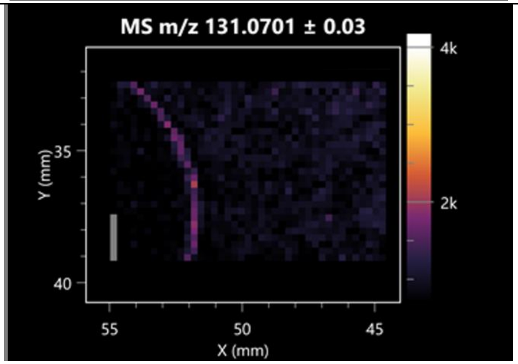	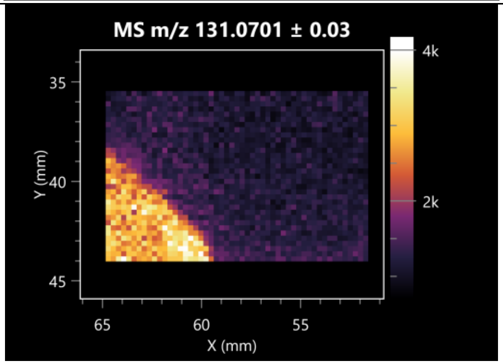	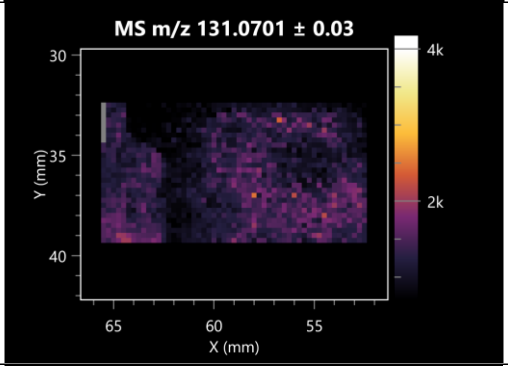
Malic acid	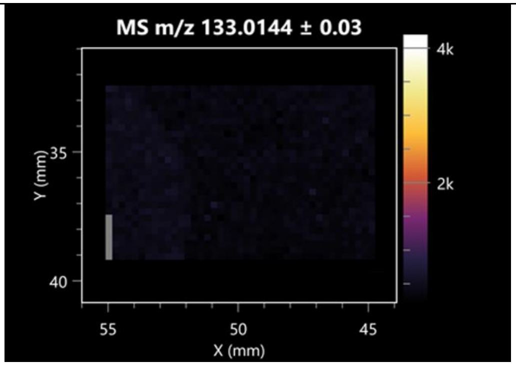	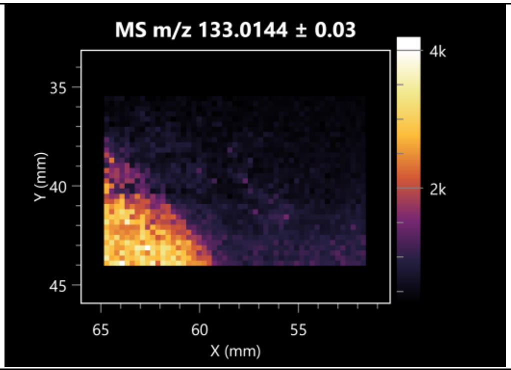	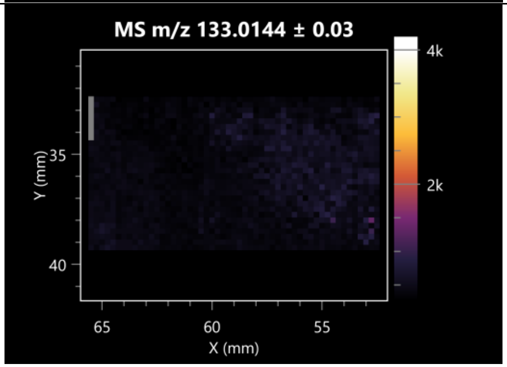

3,4-Dimethylbenzaldehyde	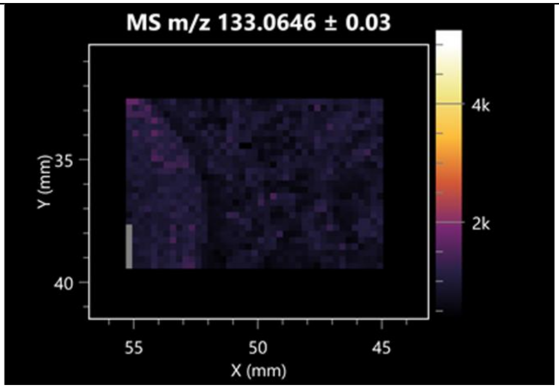	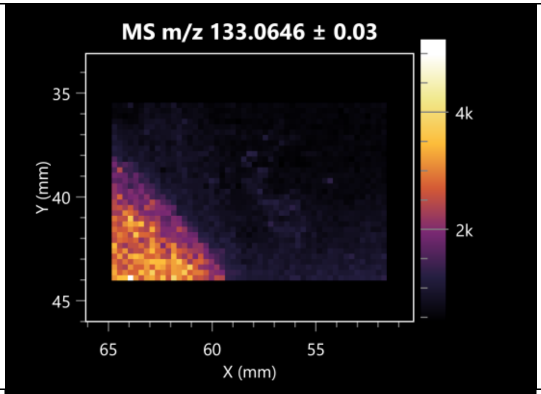	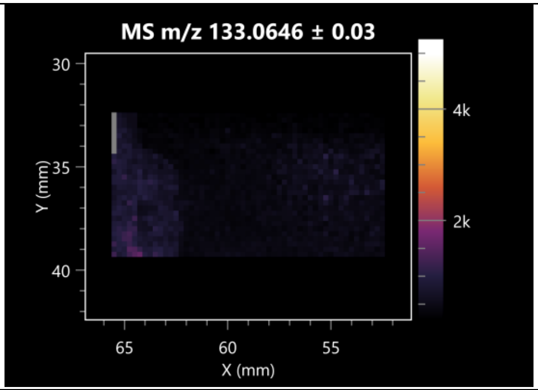
Adenine	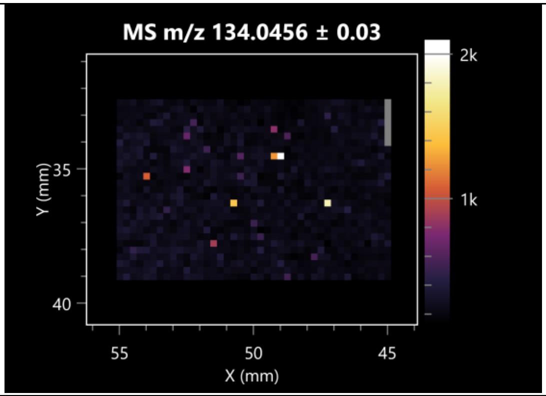	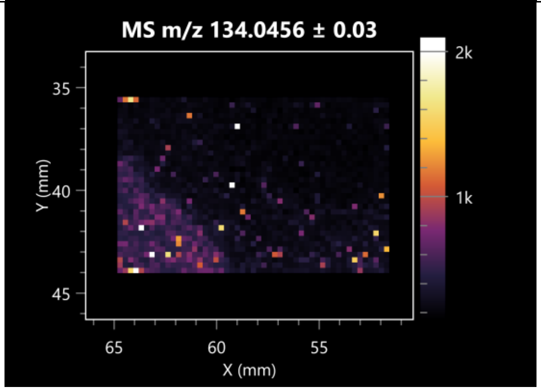	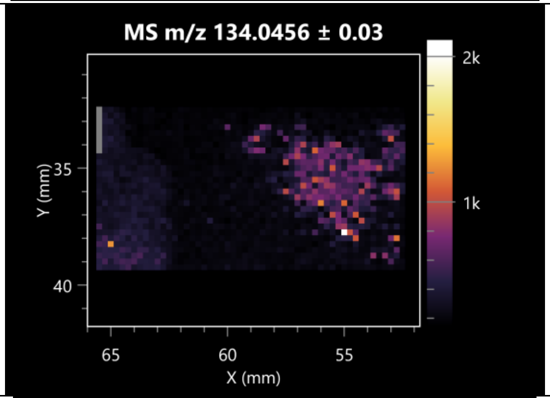
Phenylacetic acid	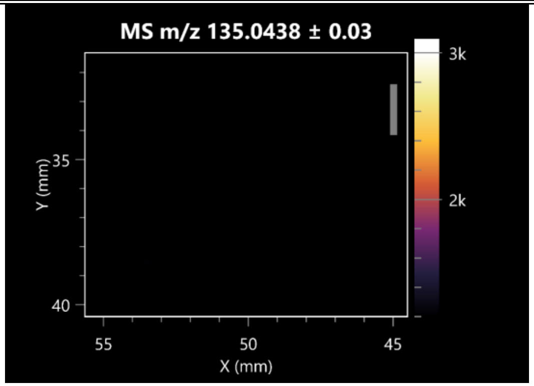	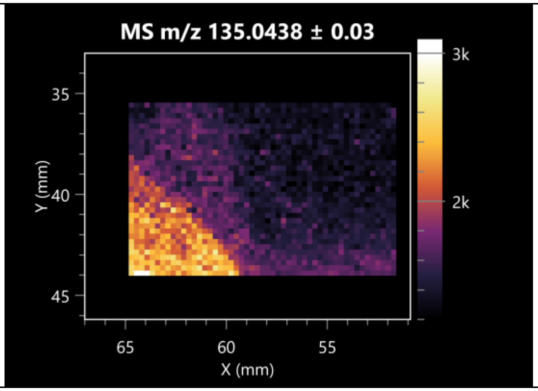	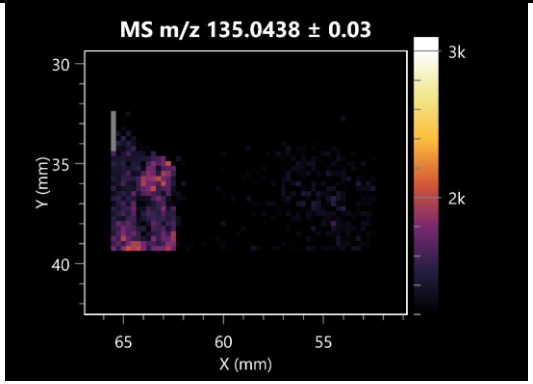

4-Hydroxyquinoline	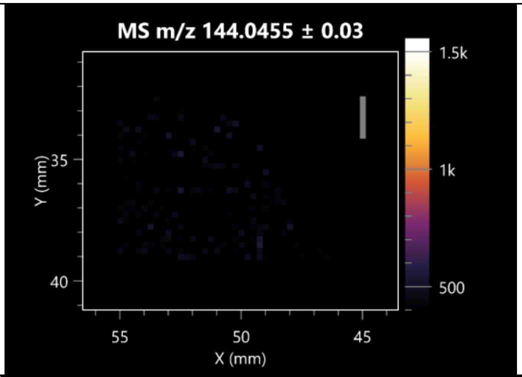	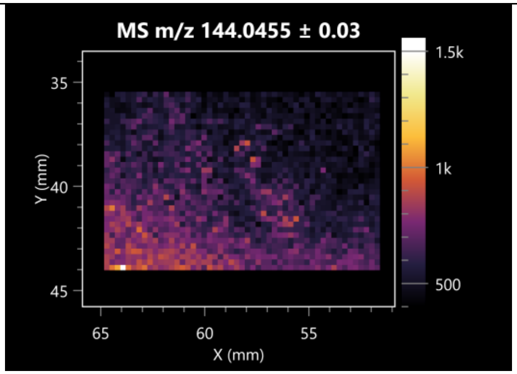	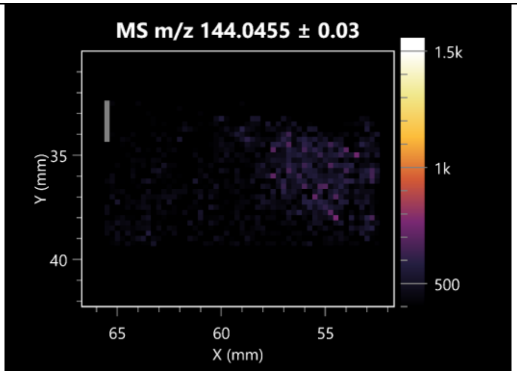
N-Methyl-D-aspartic acid	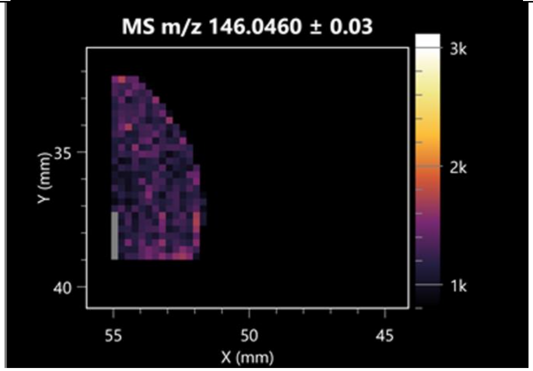	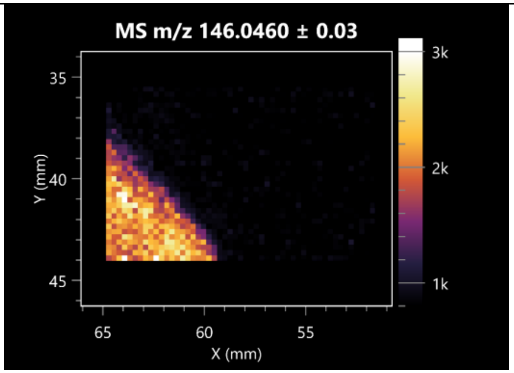	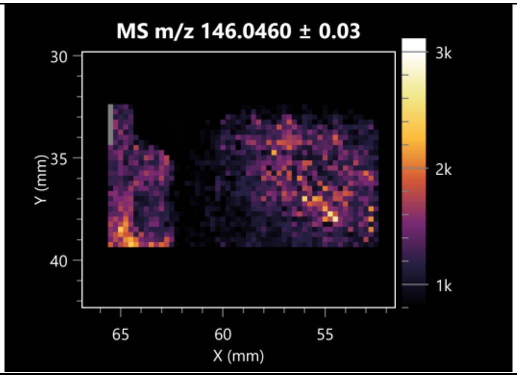
Cinnamic acid	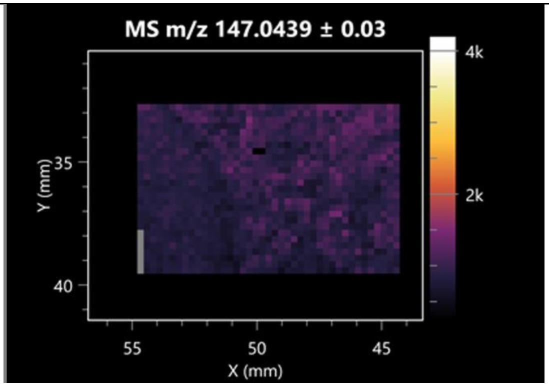	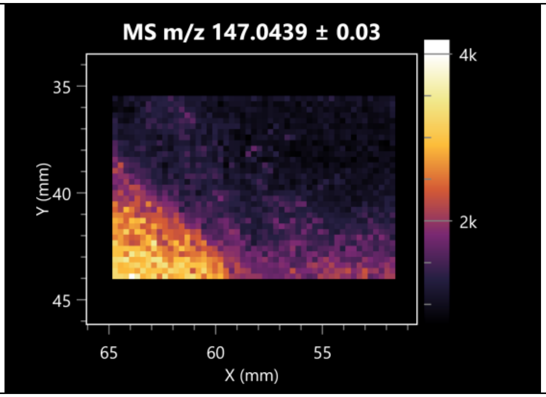	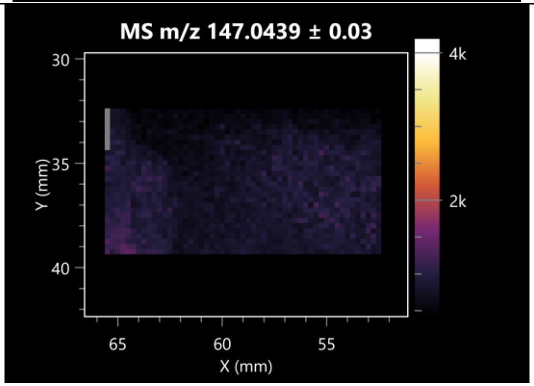

Hydrocinnamic acid	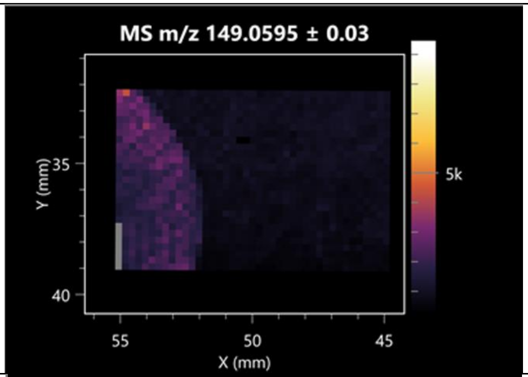	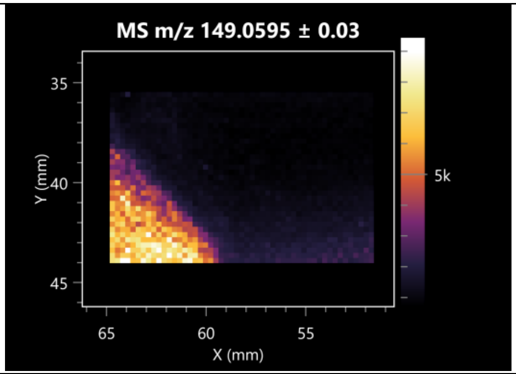	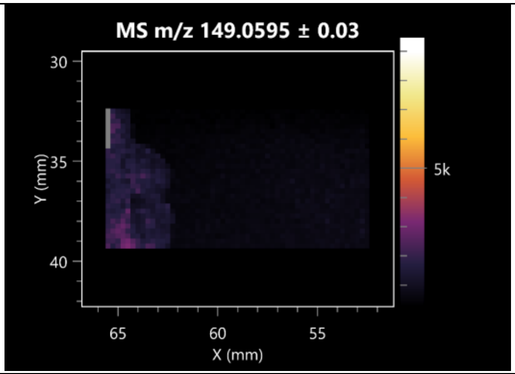
p-Anisic acid	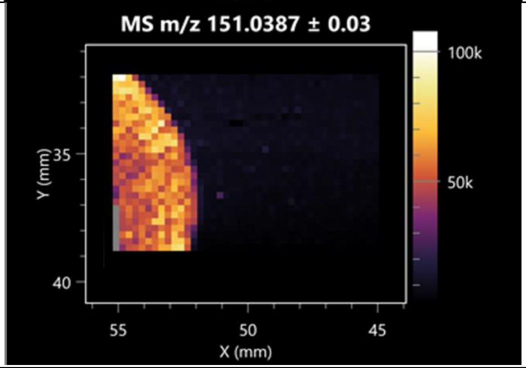	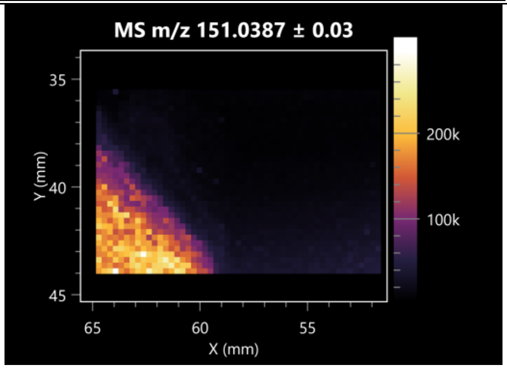	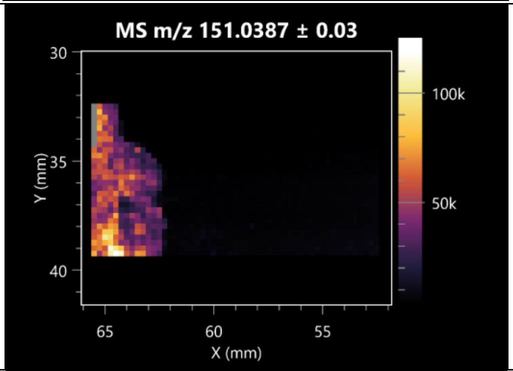
3,5-Dimethoxyphenol	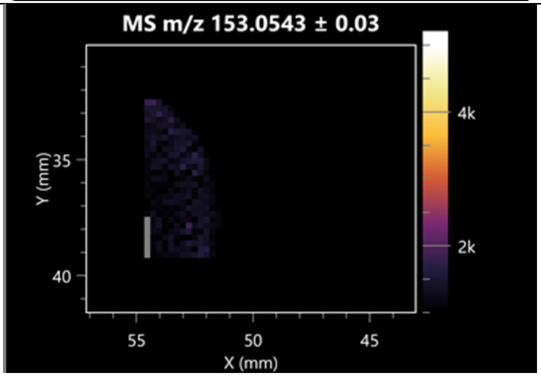	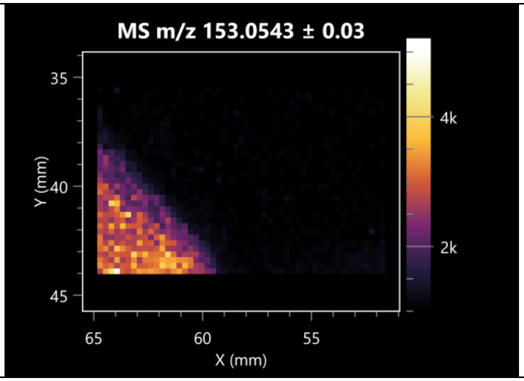	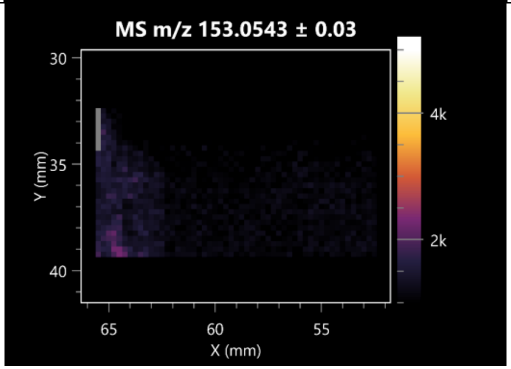

m-Coumaric acid	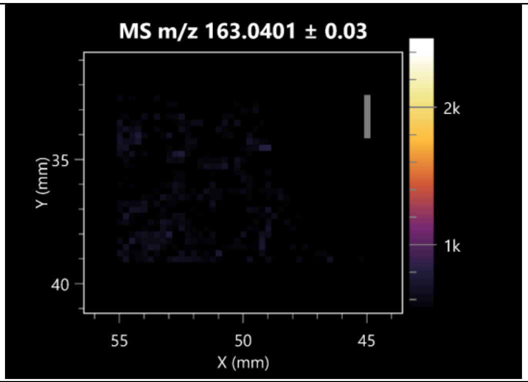	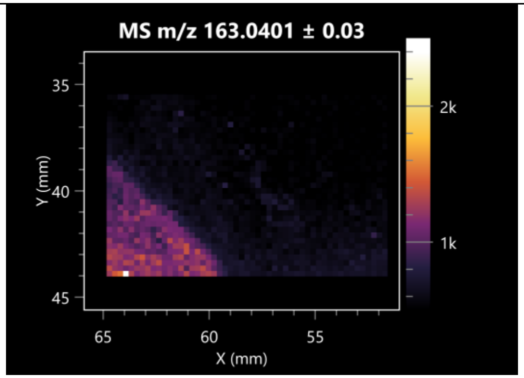	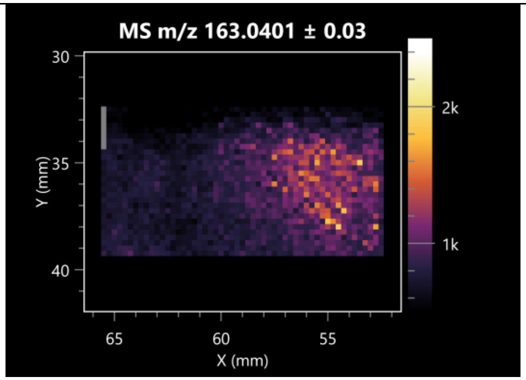
FAHFA(20:1)	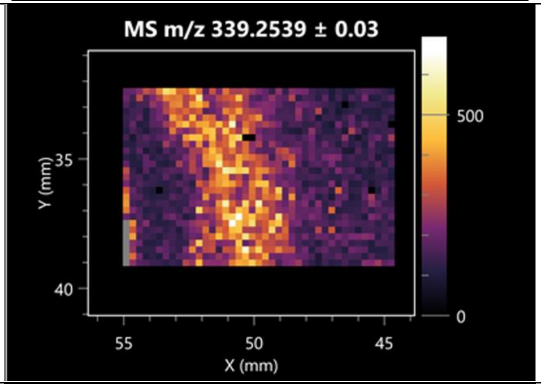	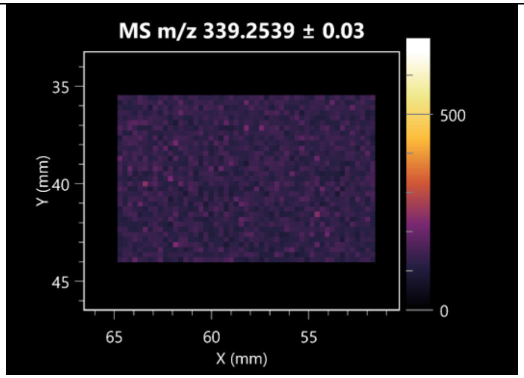	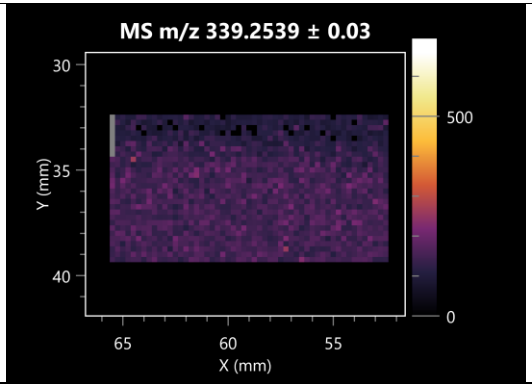
Sucrose	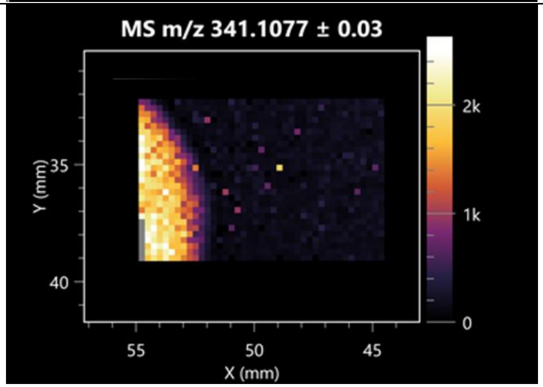	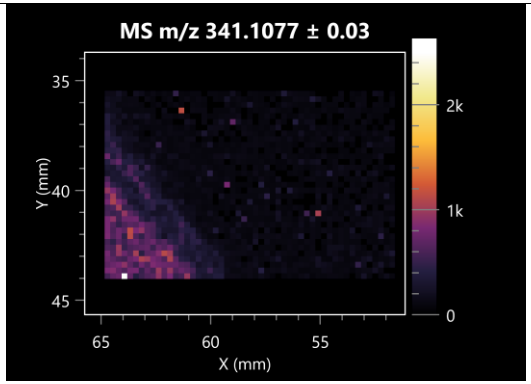	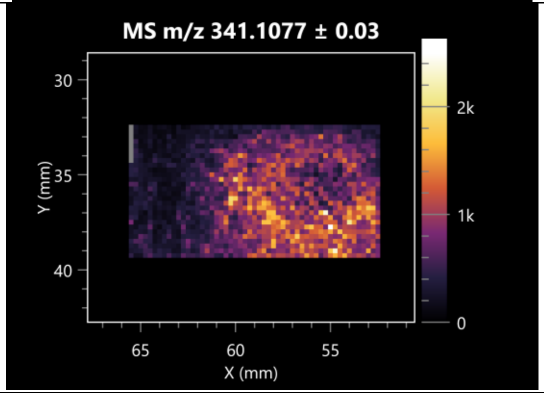

LPG(18:3)	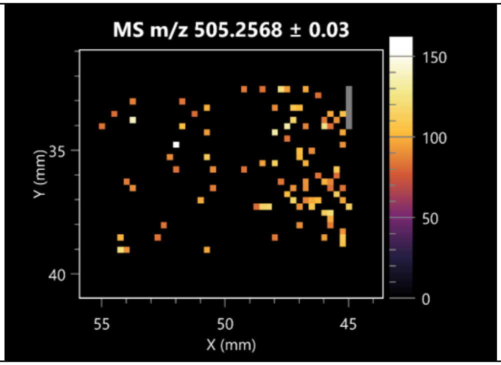MS m/z 505.2568 ± 0.03<br>Y (mm)<br>35<br>40<br>55<br>50<br>45<br>X (mm)<br>150<br>100<br>50<br>0	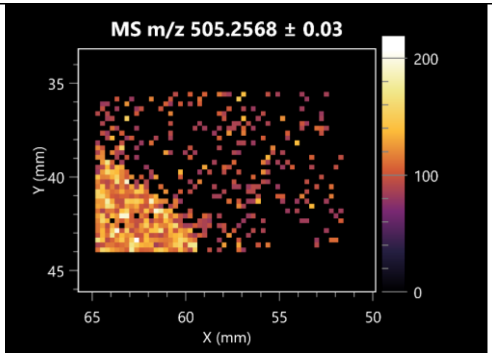MS m/z 505.2568 ± 0.03<br>Y (mm)<br>35<br>40<br>45<br>65<br>60<br>55<br>50<br>X (mm)<br>200<br>100<br>0	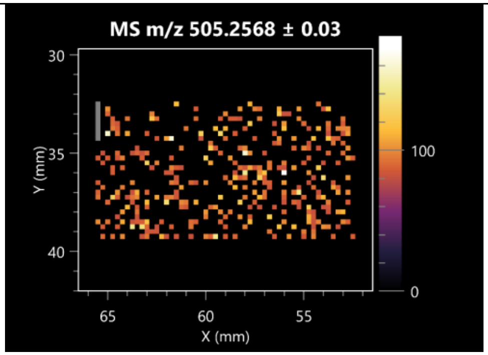MS m/z 505.2568 ± 0.03<br>Y (mm)<br>30<br>35<br>40<br>65<br>60<br>55<br>X (mm)<br>100<br>0

Table S6. Identification of chemical compounds produced by *Metschnikowia pulcherrima* yeast, strain **D4** in control sample and during co-cultivation with phytopathogenic moulds (*Rhizoctonia solani* and *Botrytis cinerea)* performed via LARAPPI/CI analysis

**Compound**	**agar gel (left) yeast strain D4 (right)**	***Botrytis cinerea* (left) yeast strain D4 (right)**	***Rhizoctonia solani* (left) yeast strain D4 (right)**
	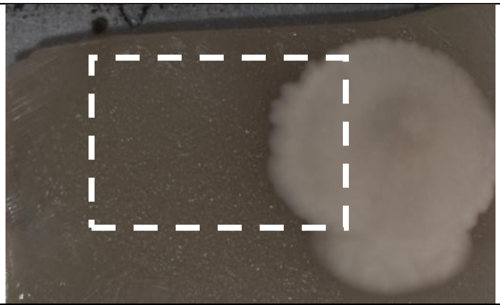	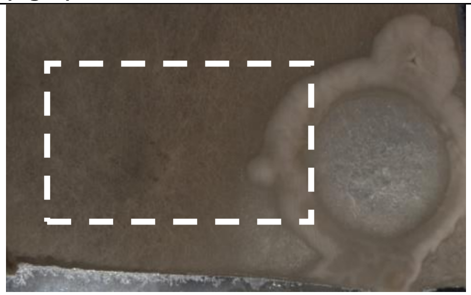	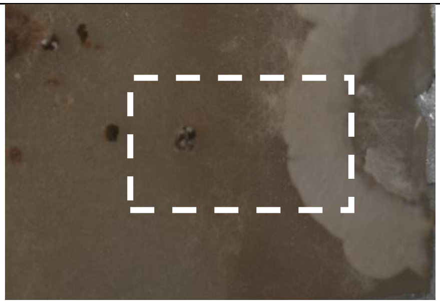
Lactic acid	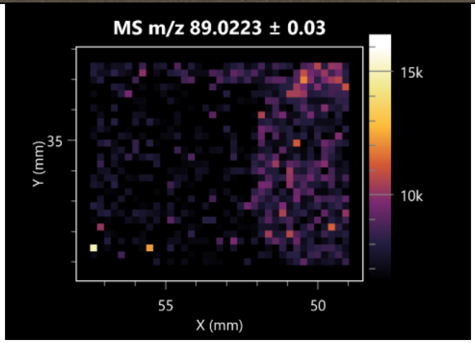	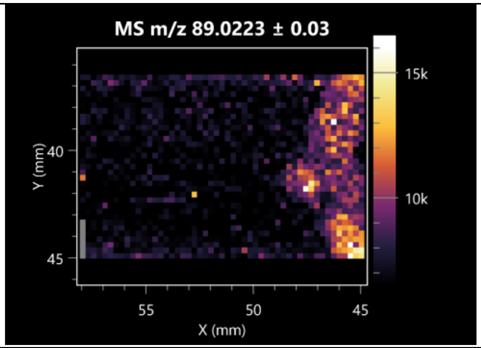	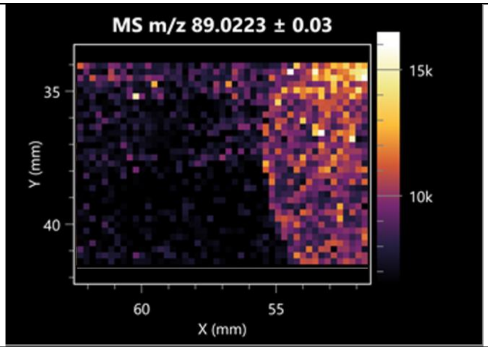
2-Ketobutyric acid	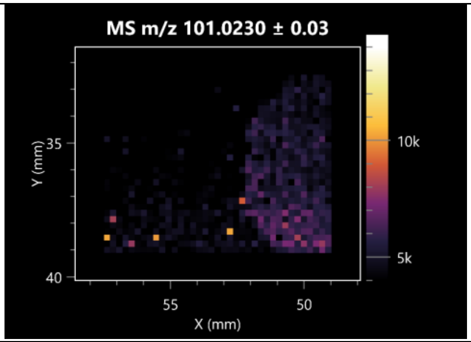	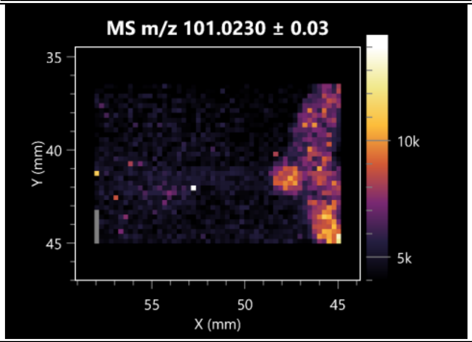	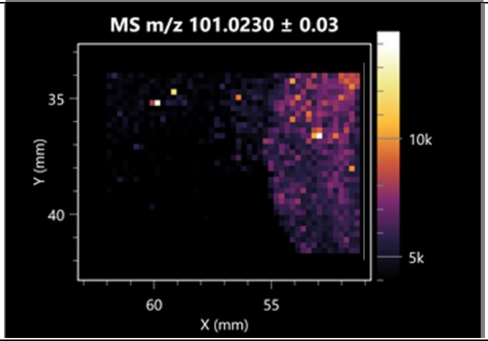

Serine	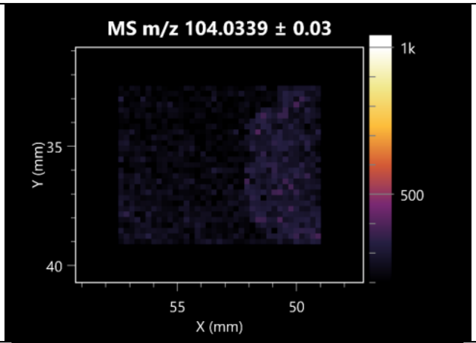	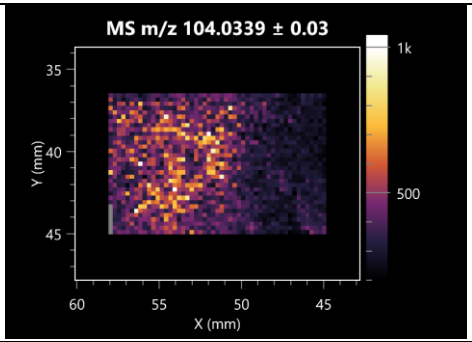	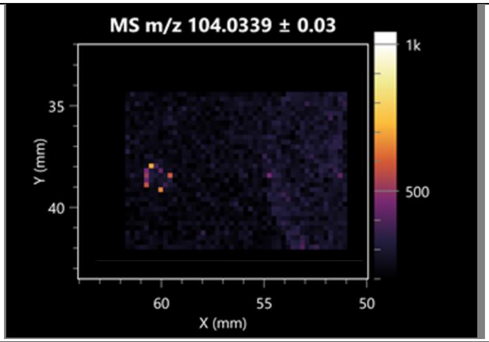
Proline	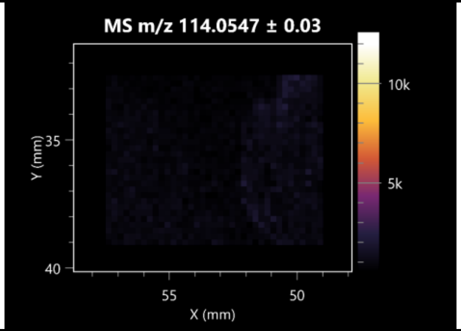	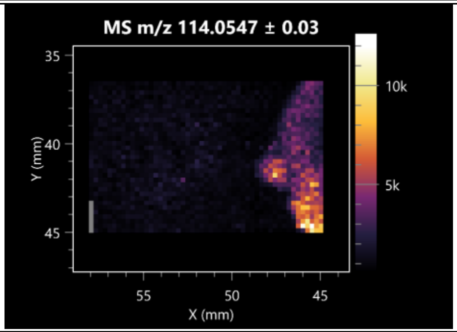	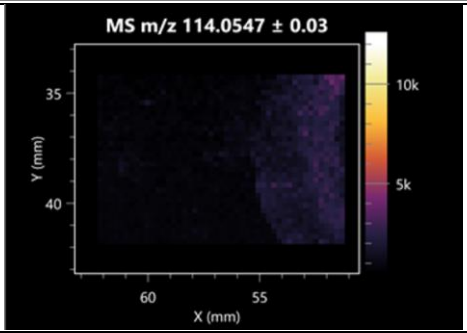
2-Hydroxy-2-methylbutyric acid	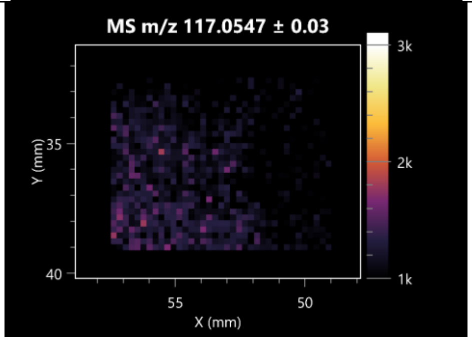	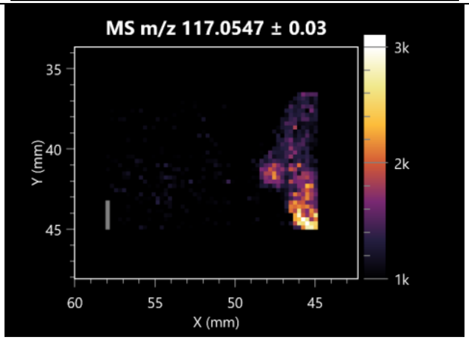	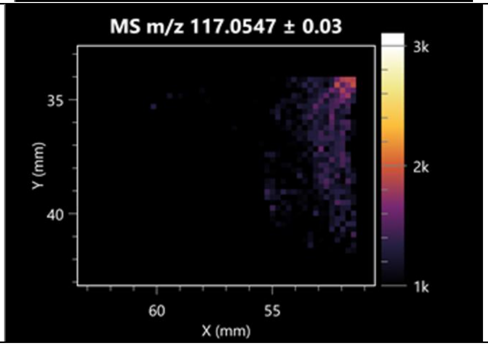

<table>
<tr><td>Threonine</td><td>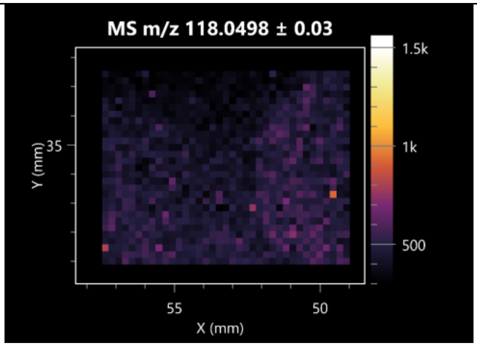
</td><td>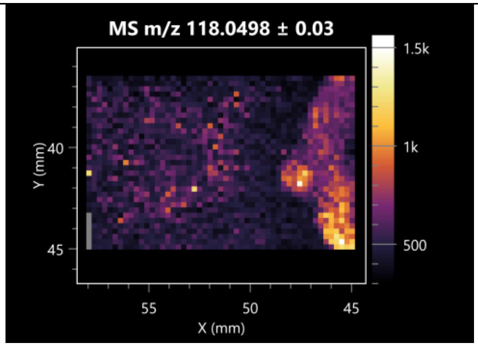
</td><td>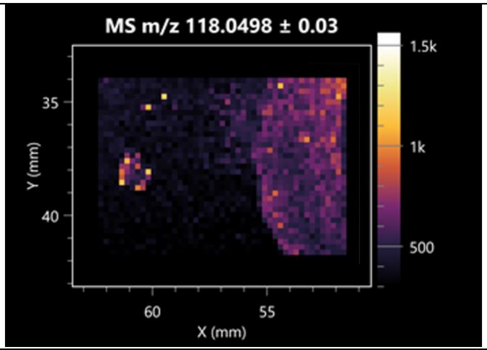
</td></tr>
<tr><td>Pipecolic acid</td><td>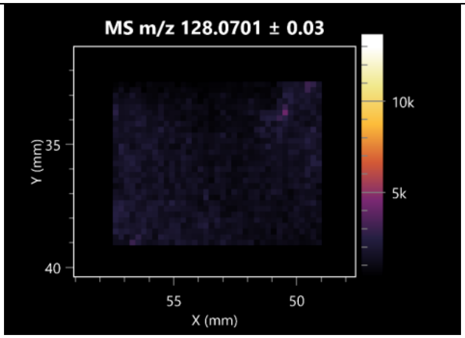
</td><td>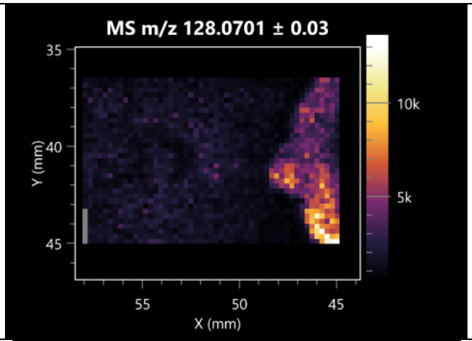
</td><td>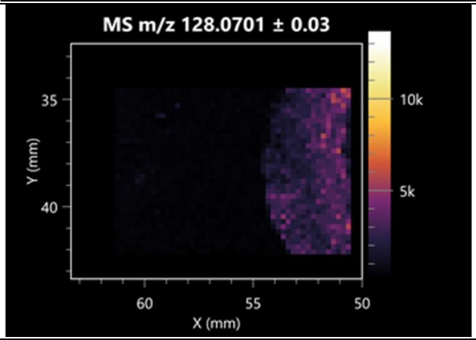
</td></tr>
<tr><td>2-Hydroxycaproic acid</td><td>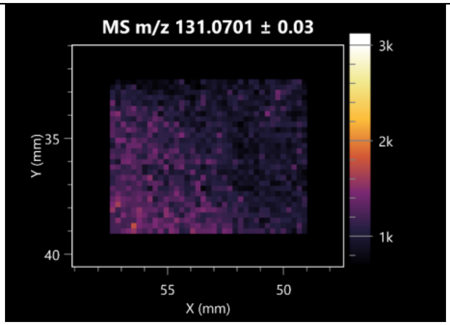
</td><td>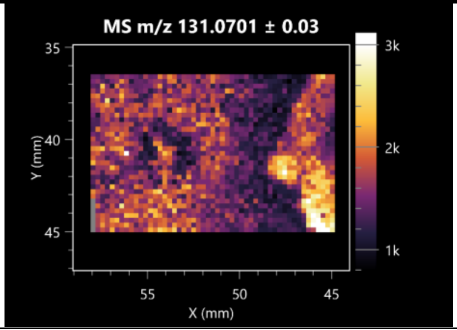
</td><td>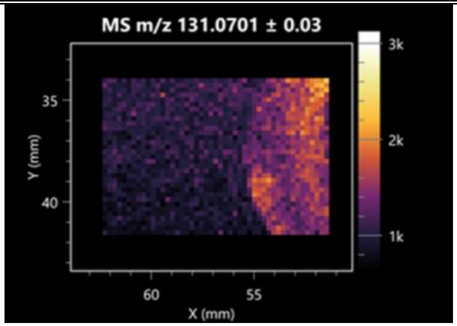
</td></tr>
</table>

Phenylacetic acid	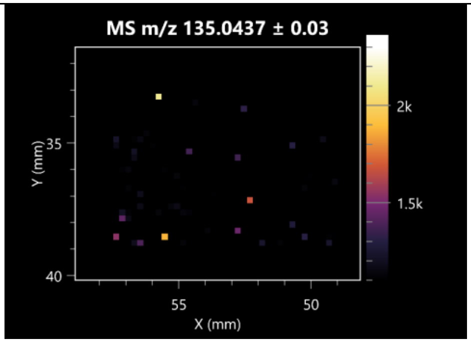	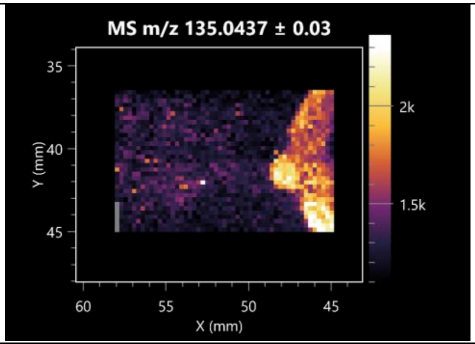	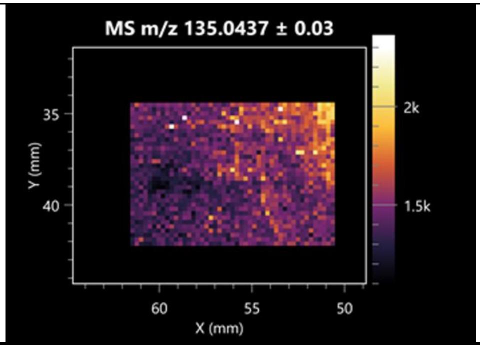
Glutamic acid	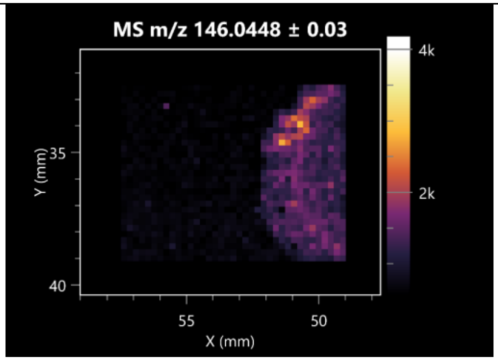	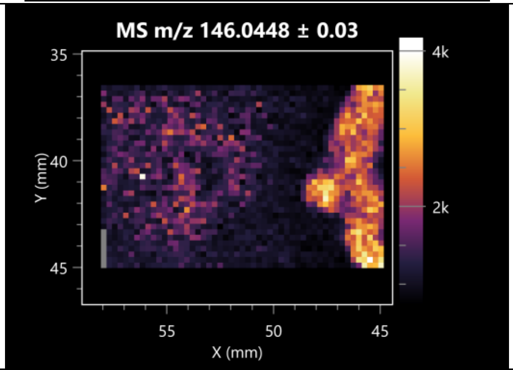	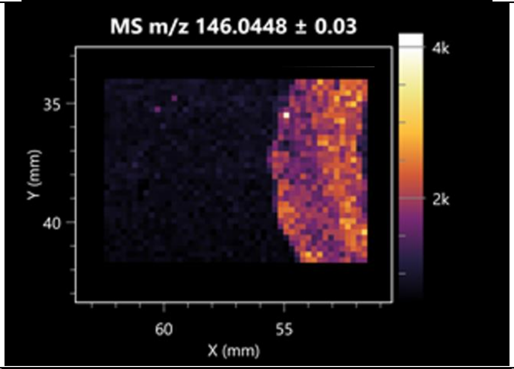
6-Methyladenine	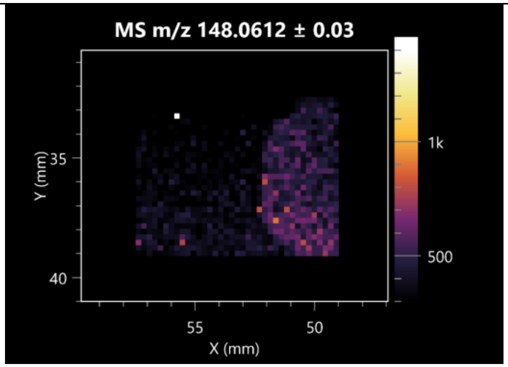	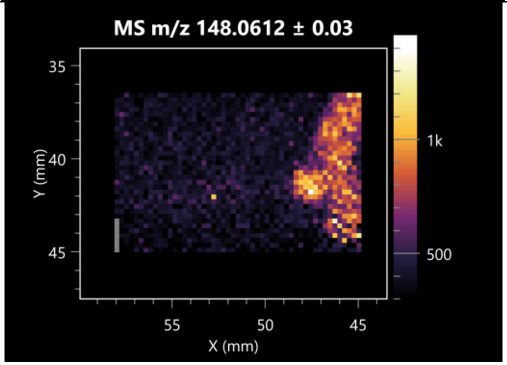	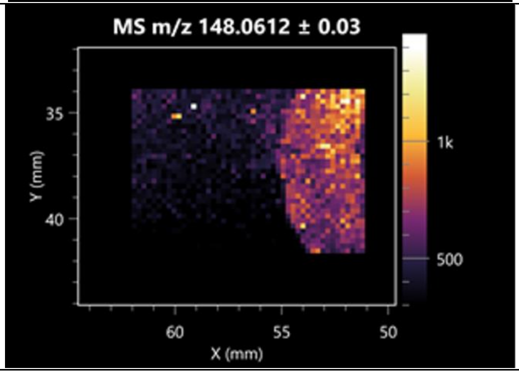

p-Anisic acid	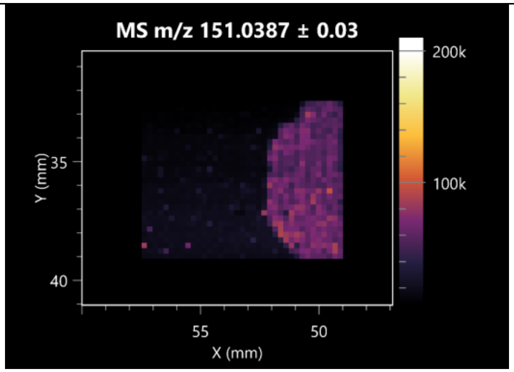	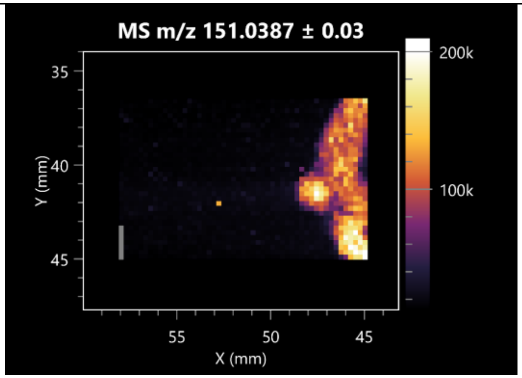	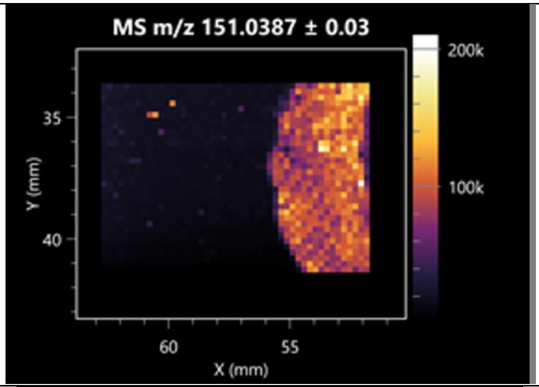
Histidine	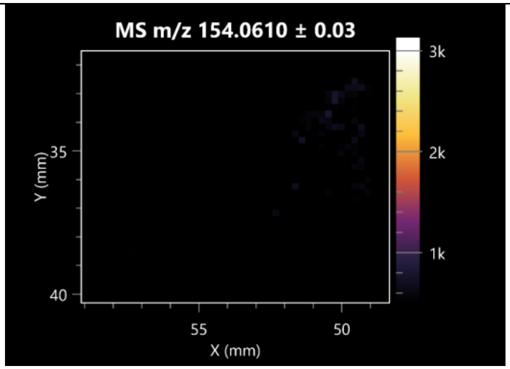	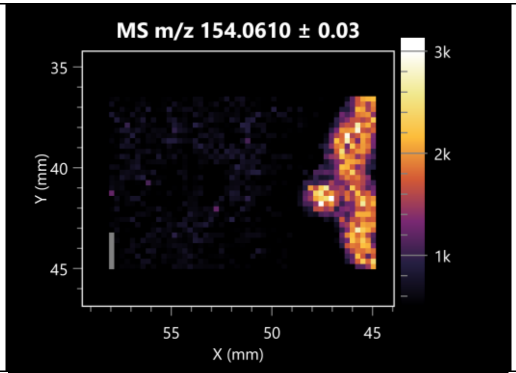	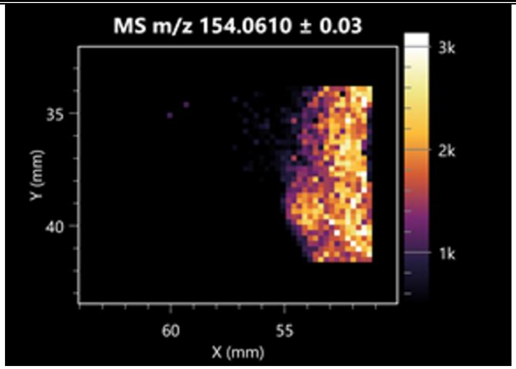
Phenylalanine	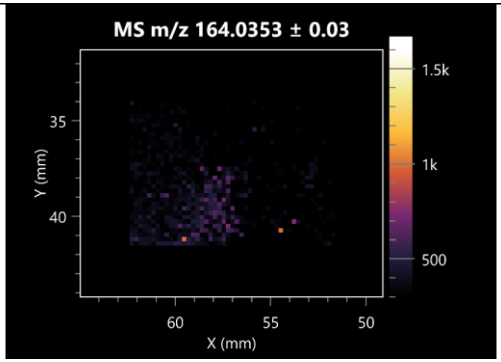	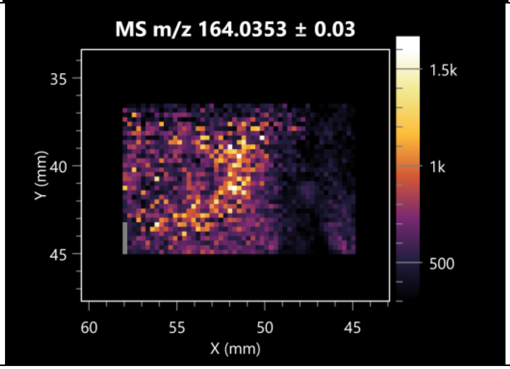	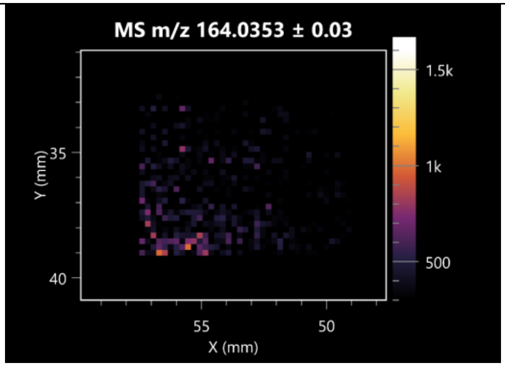

Perillic acid	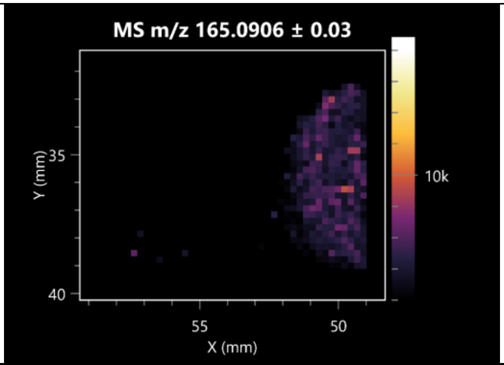	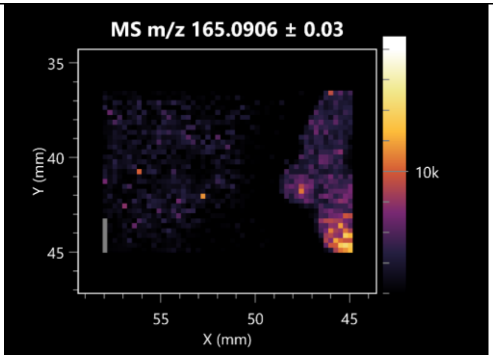	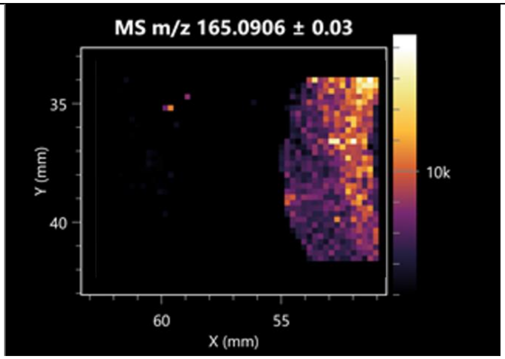
o-Tyrosine	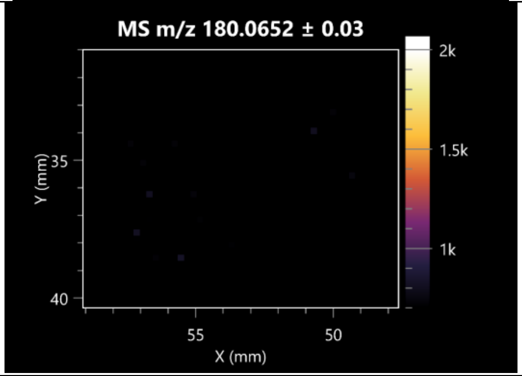	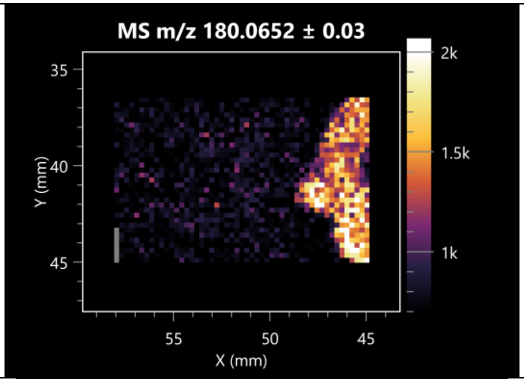	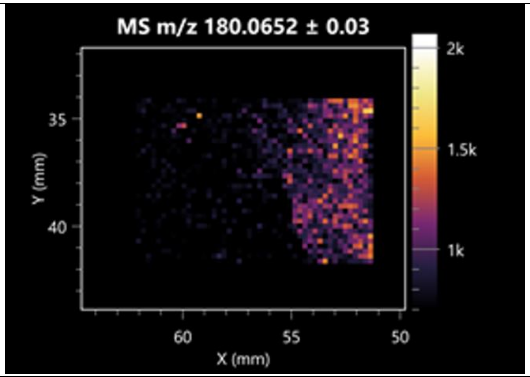
Syringic acid	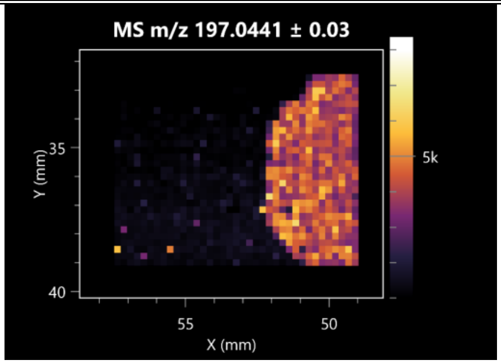	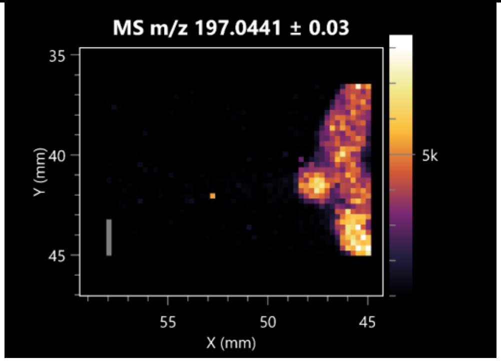	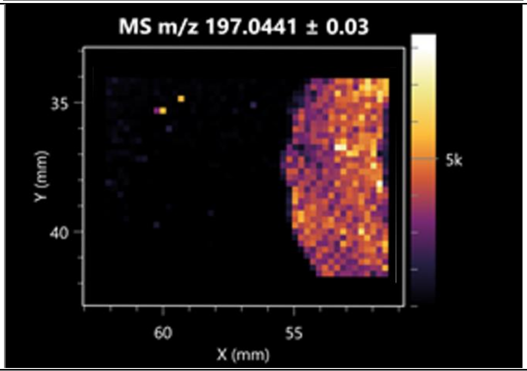

12-Hydroxyoctadecanoic acid	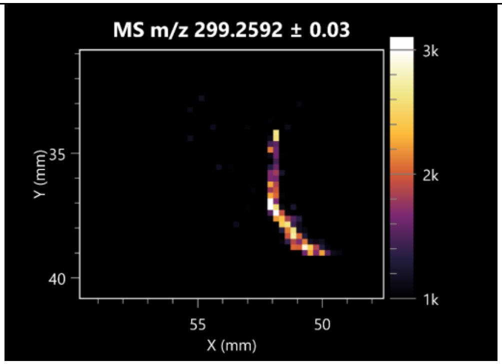	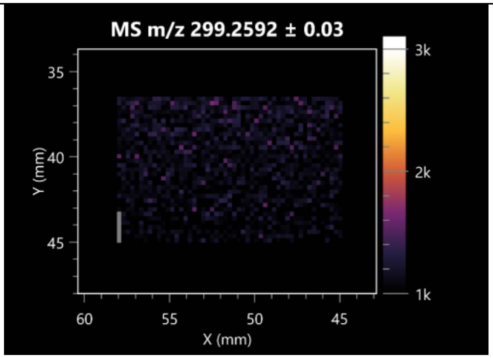	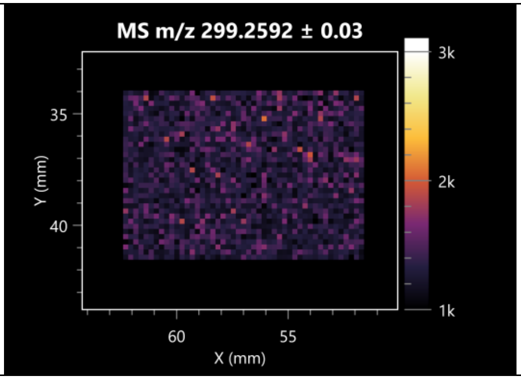

Table S7. Identification of chemical compounds produced by *Metschnikowia pulcherrima* yeast, strain **TK1** in control sample and during co-cultivation with phytopathogenic moulds (*Rhizoctonia solani, Botrytis cinerea, Alternaria alternata* and *Moinilia laxa)* performed via LARAPPI/CI analysis

**Compound**	**agar gel (left) yeast strain TK1 (right)**	***Botrytis cinerea* (left) yeast strain TK1 (right)**	***Rhizoctonia solani* (left) yeast strain TK1 (right)**	***Alternaria alternata* (left) yeast strain TK1 (right)**	***Monilia laxa* (left) yeast strain TK1 (right)**
	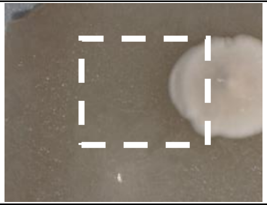	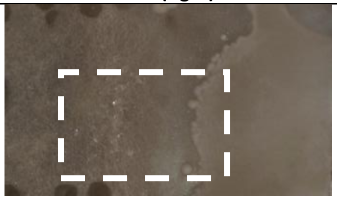	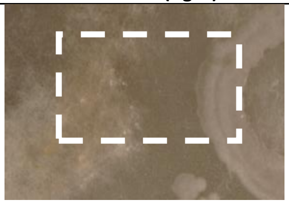	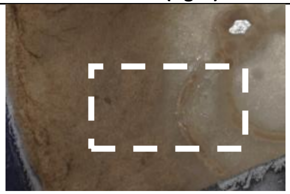	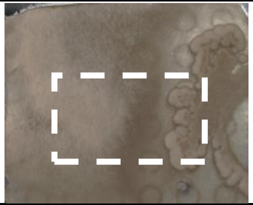
2-Ketobutyric acid	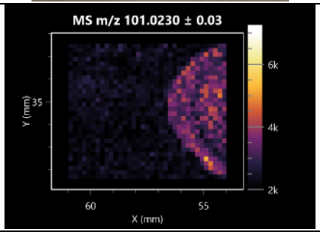	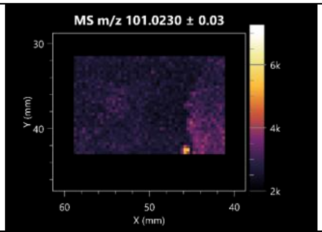	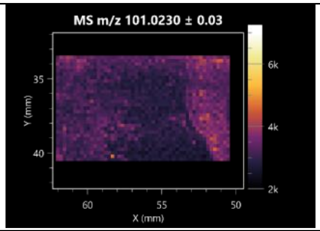	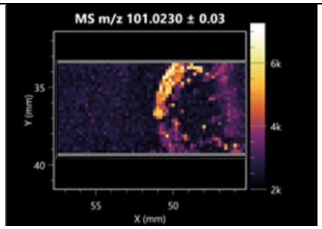	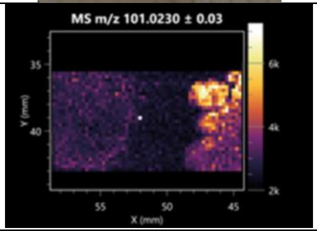
Serine	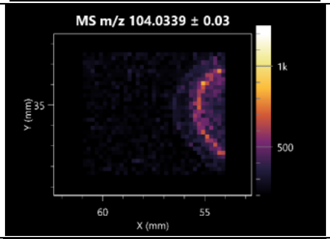	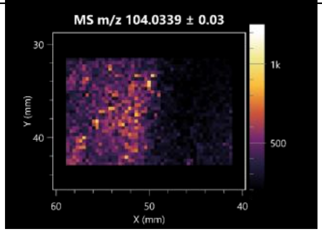	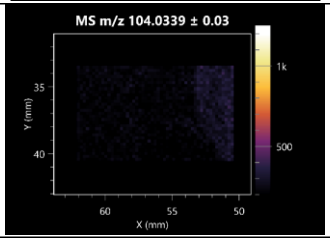	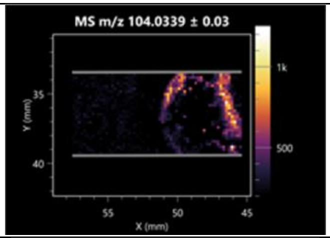	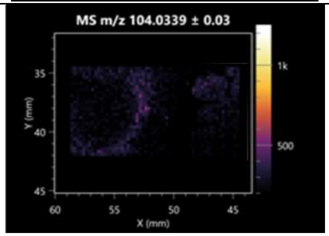
Proline	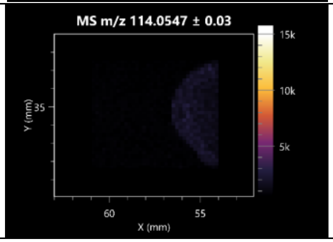	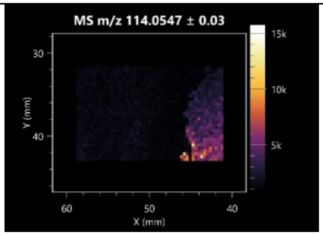	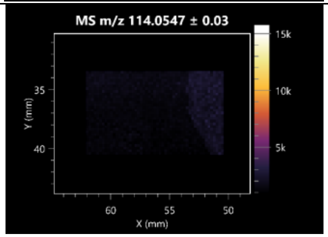	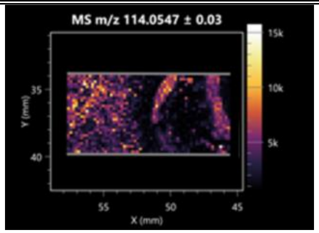	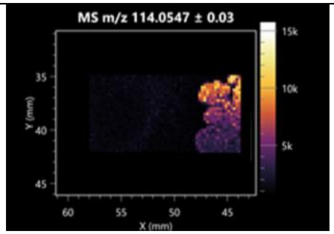

Indole	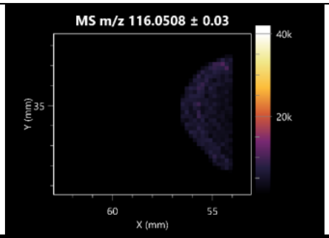	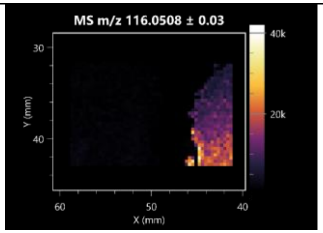	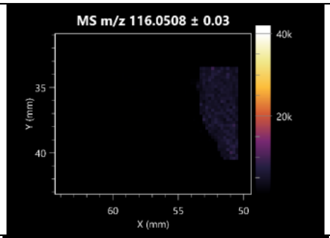	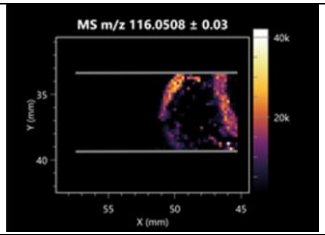	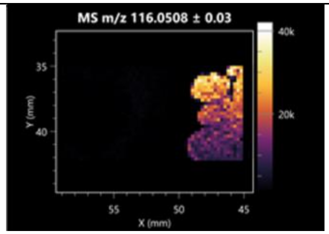
2-Hydroxy-2-methylbutyric acid	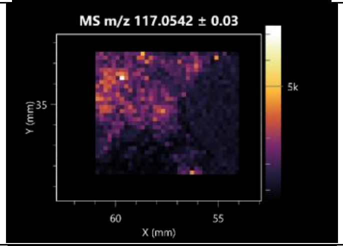	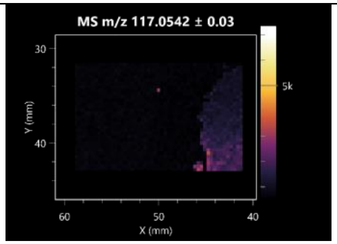	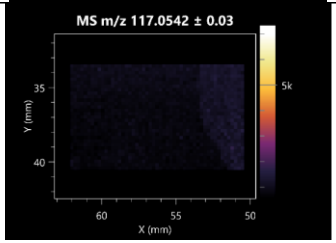	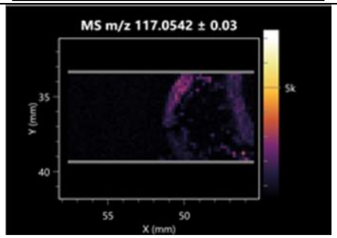	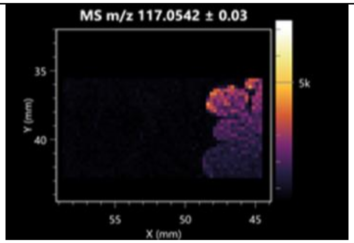
Threonine	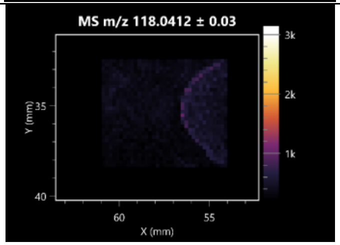	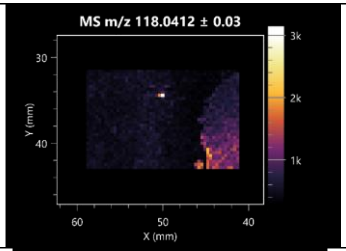	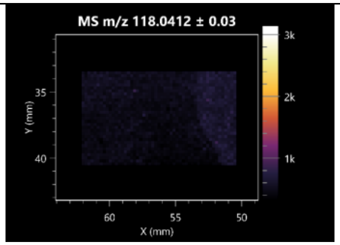	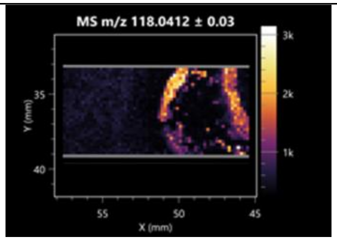	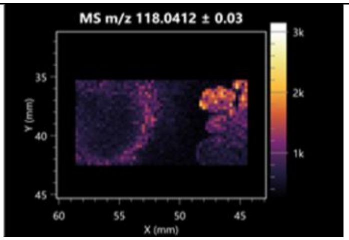
2-Hydroxycaproic acid	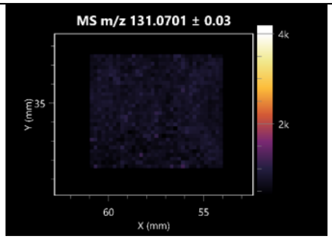	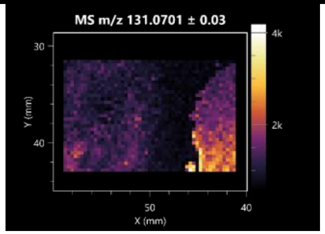	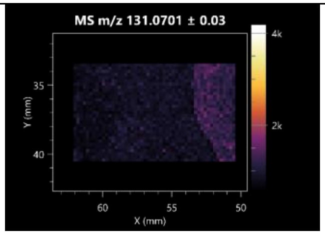	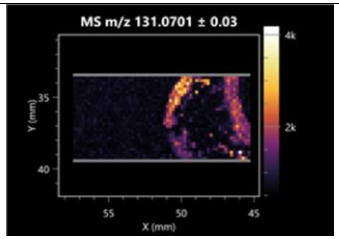	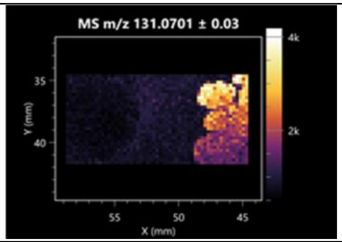
3,4-Dimethylbenzaldehyde	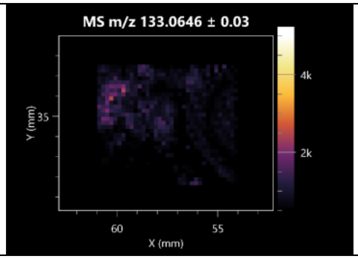	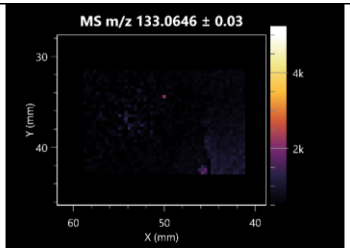	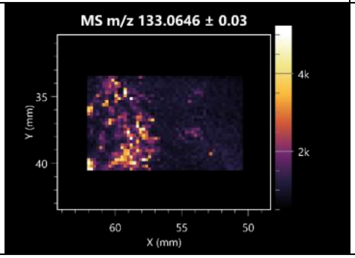	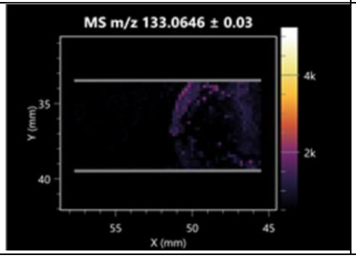	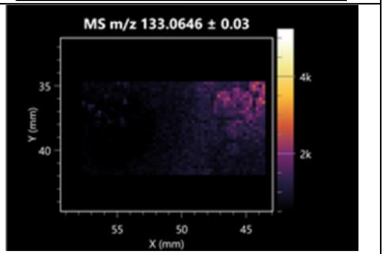

<table>
<tr><td>Adenine</td>
<td>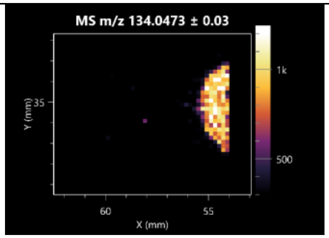</td>
<td>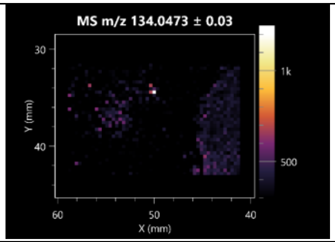</td>
<td>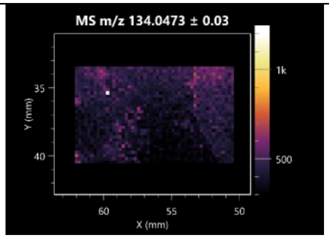</td>
<td>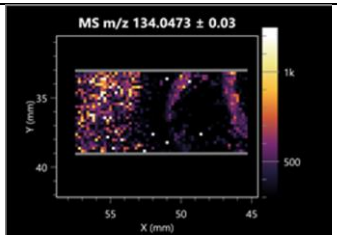</td>
<td>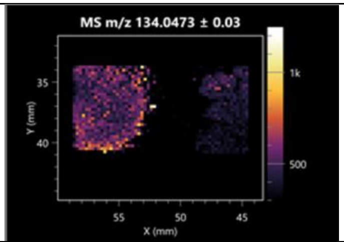</td></tr>
<tr><td>Phenylacetic acid</td>
<td>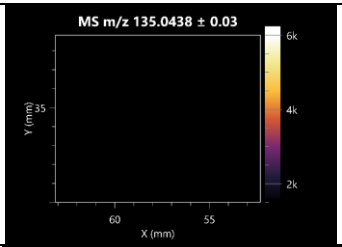</td>
<td>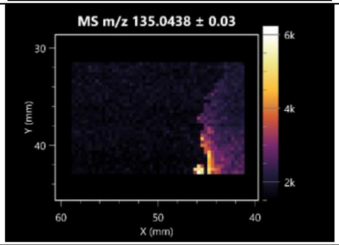</td>
<td>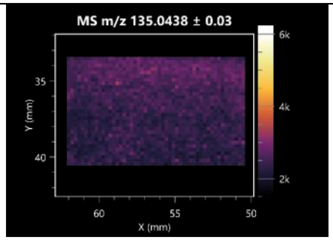</td>
<td>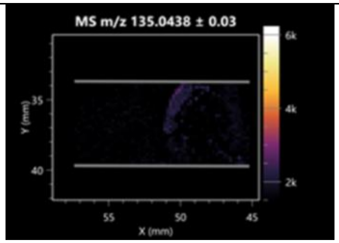</td>
<td>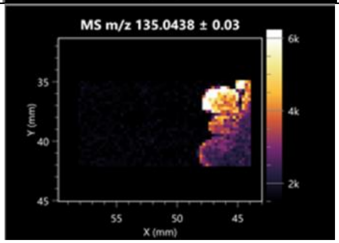</td></tr>
<tr><td>Glutamic acid</td>
<td>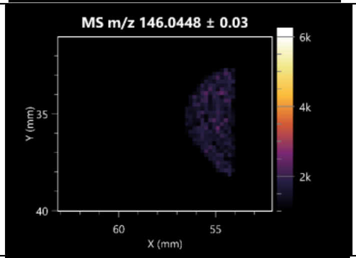</td>
<td>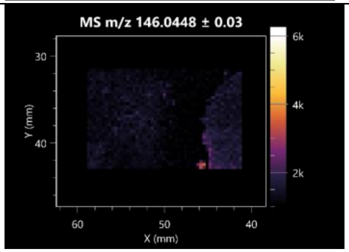</td>
<td>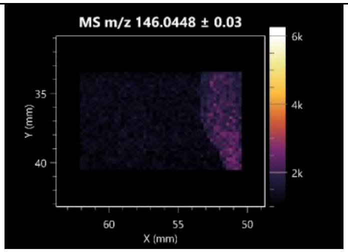</td>
<td>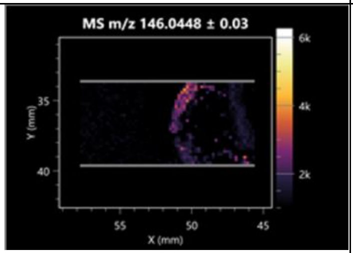</td>
<td>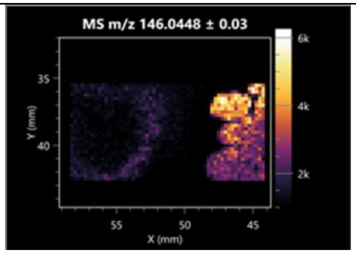</td></tr>
<tr><td>p-Anisic acid</td>
<td>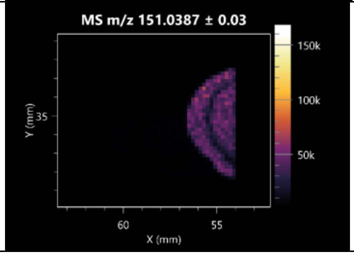</td>
<td>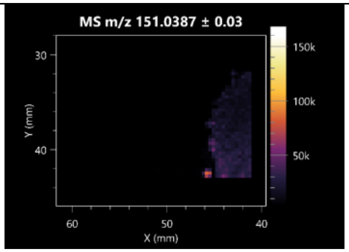</td>
<td>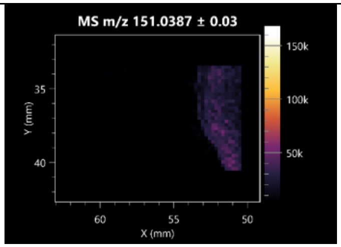</td>
<td>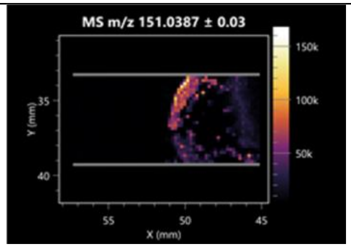</td>
<td>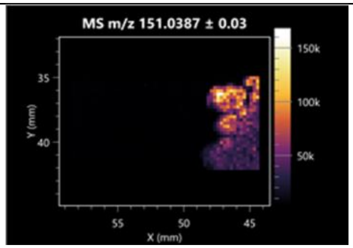</td></tr>
</table>

4-Methylcinnamic acid	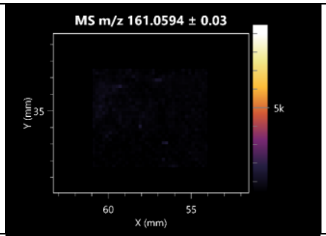	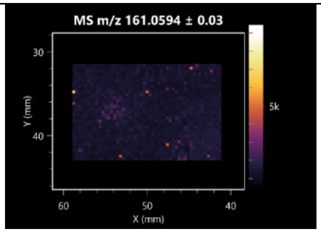	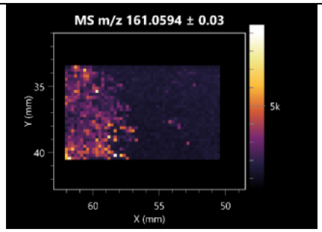	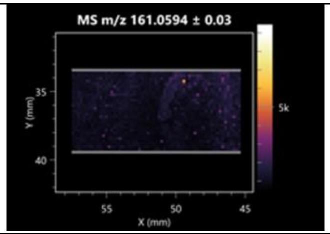	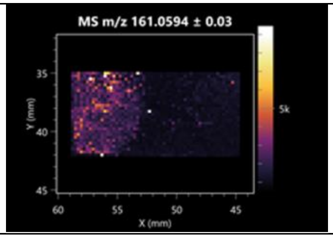
Phenylalanine	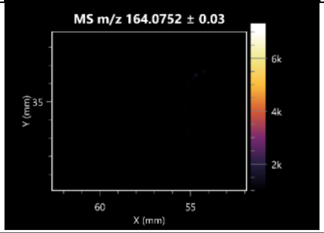	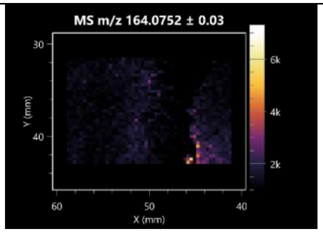	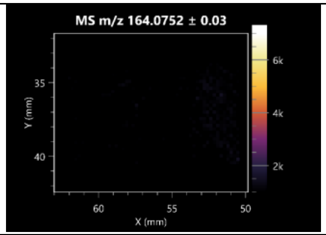	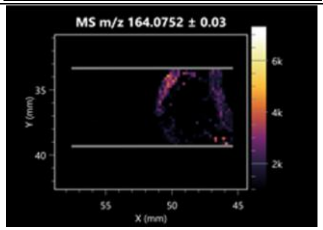	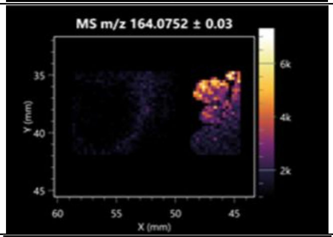
Perillic acid	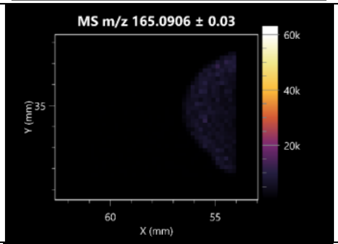	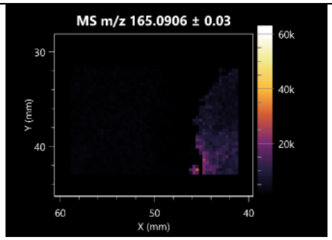	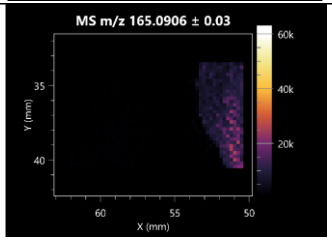	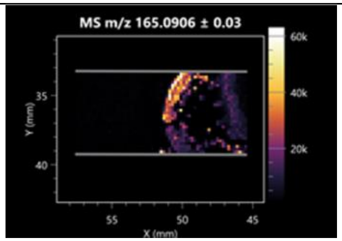	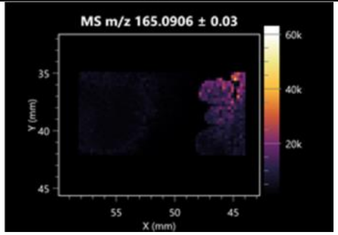
4-Pyridoxic acid	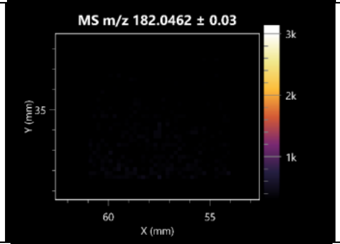	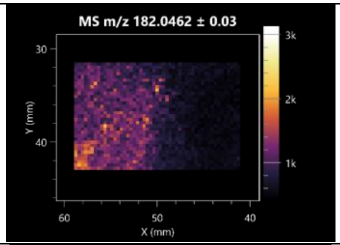	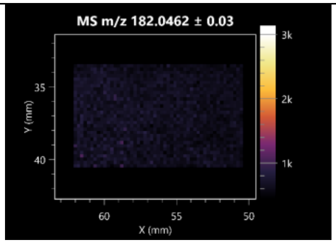	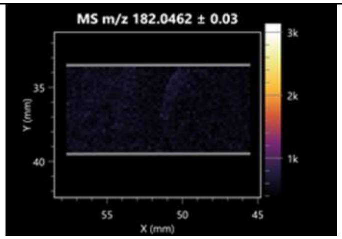	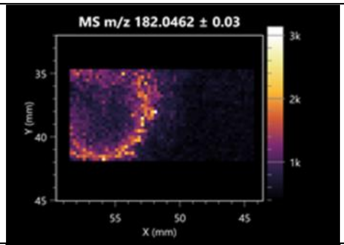
Syringic acid	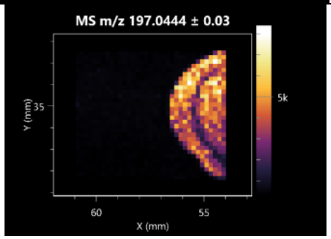	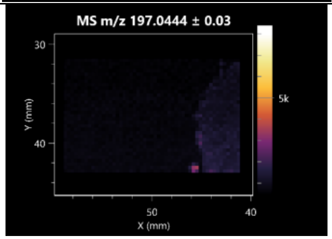	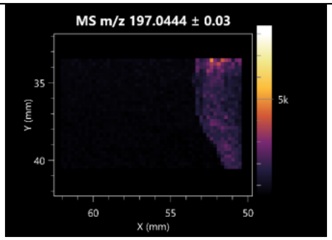	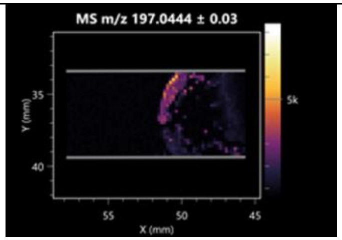	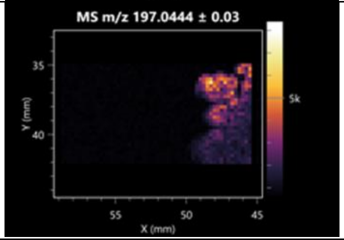

<table>
<tr><td>5-Hydroxy-L-tryptophan</td>
<td>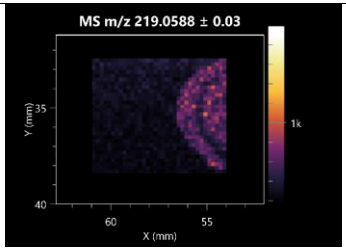</td>
<td>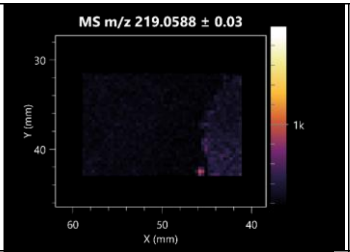</td>
<td>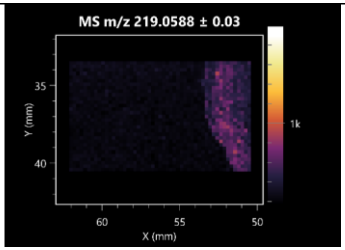</td>
<td>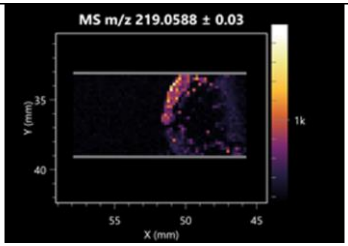</td>
<td>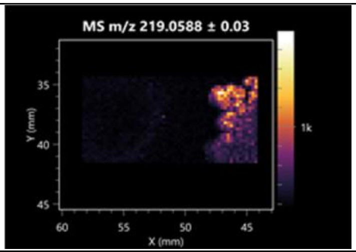</td></tr>
<tr><td>Pulcherriminic acid</td>
<td>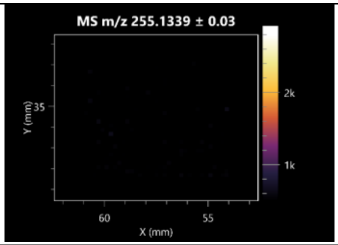</td>
<td>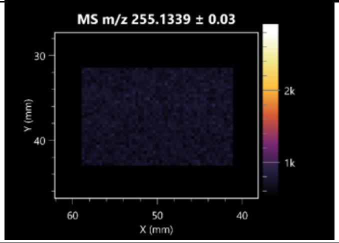</td>
<td>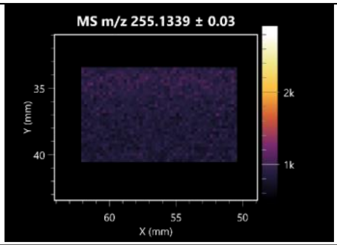</td>
<td>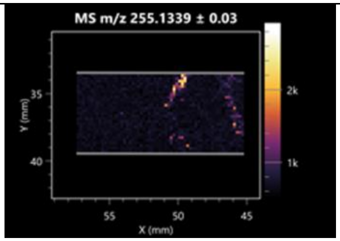</td>
<td>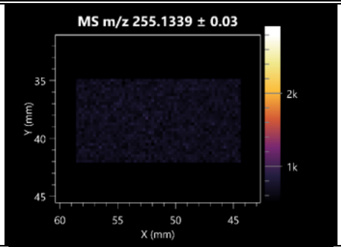</td></tr>
<tr><td>Oleic acid</td>
<td>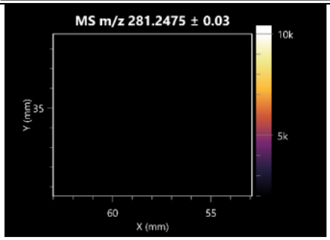</td>
<td>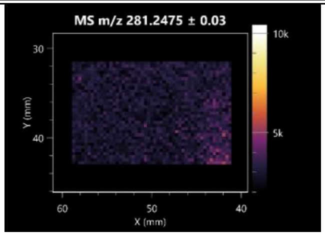</td>
<td>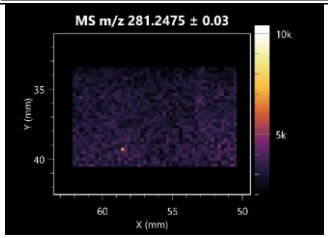</td>
<td>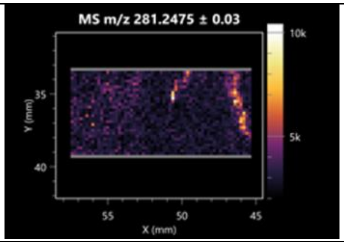</td>
<td>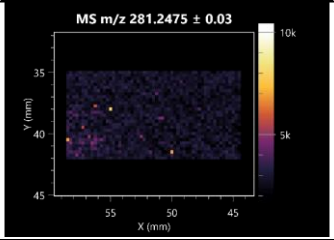</td></tr>
<tr><td>Sucrose</td>
<td>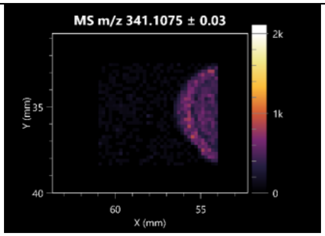</td>
<td>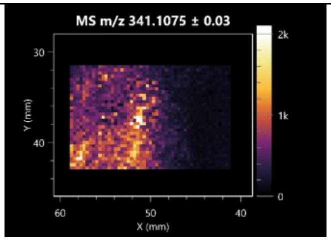</td>
<td>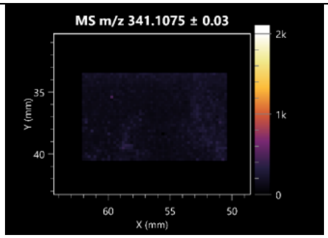</td>
<td>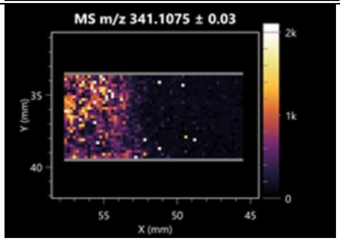</td>
<td>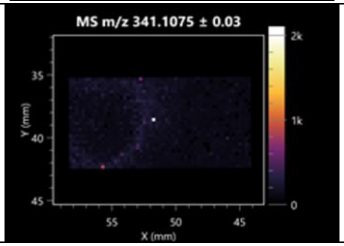</td></tr>
</table>
